# Acute contact with profibrotic macrophages mechanically activates fibroblasts via αvβ3 integrin–mediated engagement of Piezo1

**DOI:** 10.1126/sciadv.adp4726

**Published:** 2024-10-23

**Authors:** Maya Ezzo, Katrin Spindler, Jun Bo Wang, Dahea Lee, Gilbert Pecoraro, Justin Cowen, Pardis Pakshir, Boris Hinz

**Affiliations:** ^1^ Laboratory of Tissue Repair and Regeneration, Faculty of Dentistry, University of Toronto, Toronto, Ontario, Canada.; ^2^ Keenan Research Institute for Biomedical Science of the St. Michael’s Hospital, Toronto, Ontario, Canada.; ^3^ School of Life Sciences, Reutlingen University, 72762 Reutlingen, Germany.; ^4^ Princess Margaret Cancer Centre, University Health Network, Toronto, Ontario, Canada.

## Abstract

Fibrosis—excessive scarring after injury—causes >40% of disease-related deaths worldwide. In this misguided repair process, activated fibroblasts drive the destruction of organ architecture by accumulating and contracting extracellular matrix. The resulting stiff scar tissue, in turn, enhances fibroblast contraction—bearing the question of how this positive feedback loop begins. We show that direct contact with profibrotic but not proinflammatory macrophages triggers acute fibroblast contractions. The contractile response depends on αvβ3 integrin expression on macrophages and Piezo1 expression on fibroblasts. The touch of macrophages elevates fibroblast cytosolic calcium within seconds, followed by translocation of the transcription cofactors nuclear factor of activated T cells 1 and Yes-associated protein, which drive fibroblast activation within hours. Intriguingly, macrophages induce mechanical stress in fibroblasts on soft matrix that alone suppresses their spontaneous activation. We propose that acute contact with suitable macrophages mechanically kick-starts fibroblast activation in an otherwise nonpermissive soft environment. The molecular components mediating macrophage-fibroblast mechanotransduction are potential targets for antifibrosis strategies.

## Teaser

Mechanical activation by touching macrophages overrides fibroblast quiescence in soft extracellular matrix.

## INTRODUCTION

Lung fibrosis is the frequent terminal outcome of respiratory diseases, associated with a high mortality rate due to the inexorable reduction in lung function and respiratory failure. Lung fibrosis affects more than 5 million people worldwide with no effective cure (*1*). One prominent outcome and hallmark of fibrosis is the activation of fibroblasts into persistent myofibroblasts that secrete and contract collagenous extracellular matrix (ECM) (*2*, *3*). Exaggeration of these initially beneficial repair activities leads to irreversible accumulation of stiff scar tissue in the lung parenchyma that obstructs and ultimately destroys lung function (*4*, *5*).

Tissue injury, for instance to the pulmonary alveolar epithelium and ensuing inflammation, often precedes the development of fibrosis. The recruitment and activation of macrophages (Mϕ) from tissue-resident sources or circulating bone marrow–derived monocytes is part of the inflammatory response and orchestrates subsequent healing stages [reviewed in (*6*)]. During normal tissue repair, Mϕ transition from proinflammatory states into activation (polarization) states that are somewhat inconsistently described as anti-inflammatory, prorepair, proregeneration, proresolving, or profibrotic (*7*, *8*). In vitro, Mϕ at the extremes of the inflammatory-fibrotic polarization spectrum can be produced by treatment with proinflammatory agents, such as bacterial lipopolysaccharides (LPS) and/or prorepair cytokines, such as interleukin-4 (IL-4) and IL-13 (*9*, *10*). Prorepair Mϕ were shown to activate fibroblasts to express collagen type I and the contractile myofibroblast marker α-smooth muscle actin (α-SMA) (*9*, *11*, *12*). The persistence of Mϕ beyond physiological repair, dysregulated Mϕ polarization, and continued recruitment of “second wave” Mϕ into a wound tissue that already hosts fibroblasts contribute to fibrosis rather than resolution [reviewed in (*13*, *14*)]. For instance, depletion of Mϕ during the resolution phase of lung and liver repair reduces the extent of experimentally induced fibrosis, whereas Mϕ depletion in earlier healing phases exacerbates fibrosis (*15*, *16*). Thus, the timing and location of Mϕ actions and interactions with repair fibroblasts are key factors in tipping the balance between physiological repair and pathological fibrosis.

We demonstrated previously that fibroblast contractions transmitted through fibrous yet soft collagen ECM lure mechanosensitive and migratory Mϕ into an environment characteristic of early tissue repair (*17*). Numerous studies have shown that Mϕ sense stiff ECM, such as that produced by contractile fibroblasts, through different mechanoperception mechanisms, which generally drive profibrotic Mϕ phenotypes and secretory functions (*18*–*23*). From experimental studies, fibrotic cytokine “circuits” have been computationally modeled, where Mϕ provide key growth factors that activate fibroblasts, and fibroblasts provide cytokines that drive Mϕ polarization (*24*, *25*). Our own studies suggest that proximity to Mϕ is critical for myofibroblast activation over several days of coculture (*9*, *19*). Mϕ in profibrotic polarization states establish homotypic cadherin-11 adherens junctions with myofibroblasts in vitro and in fibrotic mouse and human lung tissues. The resulting prolonged direct contact keeps both cell types in proximity and establishes a niche of active profibrotic transforming growth factor–β1 (TGF-β1) signaling over several days in cocultures (*9*).

However, it is still unclear whether and how first contact with specific Mϕ polarization states can initiate fibroblast activation into cadherin-11– and α-SMA–expressing myofibroblasts. Here, we show that acute Mϕ contact formation triggers calcium (Ca^2+^) responses in fibroblasts within seconds. The Ca^2+^ response is mediated via activation of the stretch-activated membrane channel (SAC) Piezo1 on fibroblasts by Mϕ integrin–mediated membrane anchoring. Fibroblasts respond to the mechanical stimulation by Mϕ with increased contraction within minutes, higher intracellular mechanical stress, and activation of mechanosignaling pathways within hours, ultimately leading to enhanced transcription of myofibroblast-associated genes. We propose that the initiation of fibroblast contraction by Mϕ contact formation serves as a first step in scar contracture and tissue stiffening. Blocking specific Mϕ-fibroblast contact mechanisms and proteins is expected to prevent tissue contracture and myofibroblast activation and thus a valid strategy to target fibrosis.

## RESULTS

### Acute contact with profibrotic Mϕ activates fibroblasts via Yes-associated protein/transcriptional coactivator with PDZ-binding motif but not Smad signaling

We have previously shown that prolonged contact with profibrotic Mϕ activates cultured mouse lung fibroblasts into α-SMA–expressing myofibroblasts (*9*, *19*). Here, we propose that Mϕ activate fibroblasts upon first contact by initiating either TGF-β1 signaling or mechanical stimulation (or both), which are the two pivotal mechanisms of myofibroblast activation [reviewed in (*26*–*28*)]. Throughout our study, primary Mϕ were polarized by treating cultured mouse bone marrow–derived monocytes, first with Mϕ colony-stimulating factor (M-CSF) (=M_M-CSF_) followed by either LPS (=M_LPS,_ proinflammatory) or IL-4 plus IL-13 (=M_IL-4/13_, profibrotic) for 48 hours (fig. S1).

**Fig. 1.**
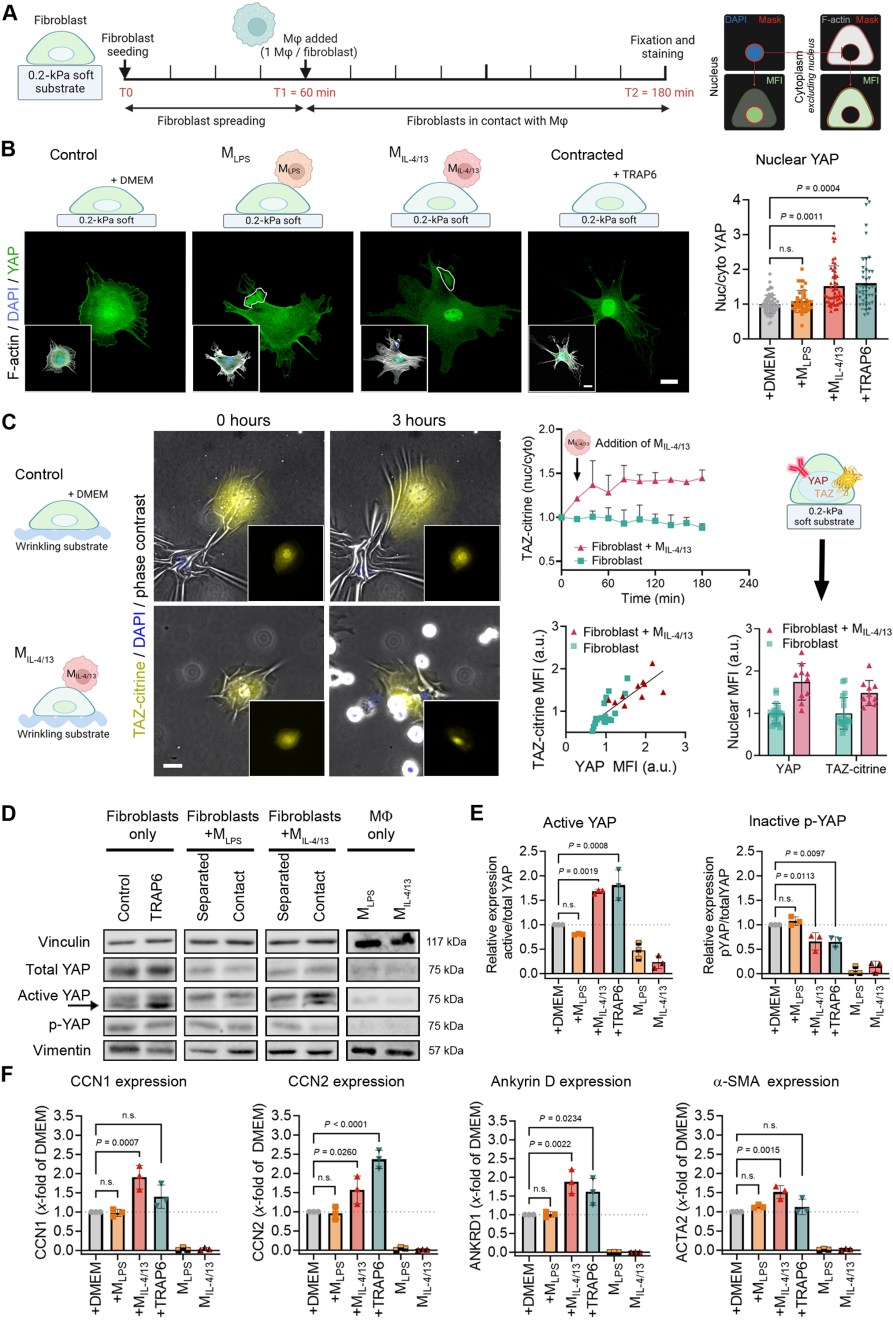
Acute Mϕ contact induces fibroblast stress on soft substrates. (**A**) Fibroblasts on 0.2-kPa soft gelatine-coated substrates were incubated with M_LPS_ or M_IL-4/13_. (**B**) After 2 hours, immunostaining for YAP (green), 4,6-diamidino-2-phenylindole (DAPI) (blue, insets), and F-actin (white, insets) was performed for control fibroblasts (+DMEM), in contact with M_LPS_ or M_IL-4/13_ (outlined), and treated with contraction agonist TRAP6. (**C**) TAZ-citrine–transfected fibroblasts (yellow) were seeded for 1 hour and then imaged for 3 hours on 0.2-kPa silicone substrates modified to observe wrinkle formation (phase contrast); M_IL-4/13_ were added after 15 min of imaging. Nuclear TAZ levels were measured, and mean fluorescence intensities (MFIs) were normalized to the first time frame. Parallel samples were stained to plot the MFIs of only nuclear or ratios of nuclear to cytoplasmic YAP over TAZ-citrine. (**D**) Correspondingly, lysates were processed for Western blotting and assessed for expression of total YAP, active YAP (arrow, expected molecular weight), and phosphorylated YAP (p-YAP) with vinculin and vimentin as loading controls. To have comparable protein loading to Mϕ-fibroblast cocultures (“contact”), fibroblasts and Mϕ were cultured alone for the same time but mixed just before lysis (“separated”). (**E**) Western blot band intensities for active YAP and p-YAP, normalized to total YAP. (**F**) Quantitative reverse transcription polymerase chain reaction was performed with cells grown in the same conditions to measure YAP target genes, normalized to *Gapdh*. All column graphs show mean values (±SD) after normalization to fibroblasts grown alone in control DMEM. Experiments were performed with cells from three different mice, every data point representing one biological repeat, except in (B) and (C), where every data point represents one of 40 to 50 fibroblasts. All scale bars, 20 μm. Statistical significance was calculated using repeated measures analysis of variance (ANOVA), and Fisher’s least significant difference (LSD) post hoc analysis with significance reached with *P* < 0.05 and “n.s.” indicating not significant.

To test whether acute Mϕ contact elicits a mechanical stress response in fibroblasts, we tracked the transcription factors Yes-associated protein (YAP) and transcriptional coactivator with PDZ-binding motif (TAZ) as indicators of mechanical myofibroblast activation (*29*). Primary mouse lung fibroblasts were first spread for 1 hour on gelatin-coated 0.2-kPa “soft” silicone substrates to maintain low-baseline mechanical myofibroblast activation (*30*) (Fig. 1A). Either M_LPS_ or M_IL-4/13_ was then added in a 1:1 Mϕ:fibroblast ratio; Dulbecco’s modified Eagle’s medium (DMEM) without Mϕ was used as negative control, and thrombin receptor–activating peptide 6 (TRAP6) was added as a positive control to induce fibroblast cytoskeletal stress (*31*). The levels of nuclear YAP were measured after an additional 2 hours as an indicator of fibroblast mechanical stress (*32*) (Fig. 1A). Contact with M_IL-4/13_ and treatment with TRAP6 elicited 1.54- and 1.65-fold increases of nuclear YAP, respectively, compared to fibroblasts alone; adding M_LPS_ had no effect (Fig. 1B). YAP antibody staining was validated by live tracking of citrine-labeled fluorescent constructs of TAZ that translocated to the nucleus of fibroblasts within 60 min after contact with M_IL-4/13_; costaining of TAZ-citrine–transfected fibroblasts with anti-YAP antibodies corroborated that YAP and TAZ respond to M_IL-4/13_ contact (Fig. 1C and movie S1).

Activation of fibroblast YAP signaling by M_IL-4/13_ contact was further quantified from Western blots performed with fibroblast and Mϕ lysates obtained after 2 hours of direct contact (Fig. 1D, “contact”), or mixed after culturing them apart from each other as control (Fig. 1D, “separated”). Equal protein loading was first confirmed using vinculin and vimentin as housekeeping proteins; active and nonactive YAP were subsequently normalized to total YAP (Fig. 1, D and E). Normalized levels of nonphosphorylated active YAP were 1.68 times higher and phosphorylated inactive YAP 0.66 times lower in fibroblasts contacted by M_IL-4/13_ compared to the mix of separately grown fibroblasts and M_IL-4/13_; no changes in YAP activation were measured in fibroblasts in contact with M_LPS_ (Fig. 1, D and E). M_IL-4/13_-induced changes in YAP phosphorylation and localization were accompanied by enhanced expression of myofibroblast-associated genes known to be transcriptionally regulated by YAP (*29*, *32*). Fibroblasts in contact with M_IL-4/13_ exhibited increased expression of *Ccn1* (1.91-fold), *Ccn2* (1.56-fold), *Ankrd1* (ankyrin repeat domain 1) (1.88-fold), and *Acta2* (α-SMA) (1.51-fold), respectively (Fig. 1F), compared to fibroblasts alone or in contact with M_LPS_. M_IL-4/13_ and M_LPS_ monocultures had no noticeable levels of nuclear YAP (Fig. 1, B and C), YAP protein (Fig. 1, D and E), or YAP target gene expression (Fig. 1F). These results support that acute contact with “profibrotic” M_IL-4/13_ but not “proinflammatory” M_LPS_ initiates fibroblast-to-myofibroblast activation within minutes through YAP/TAZ signaling.

**Fig. 2.**
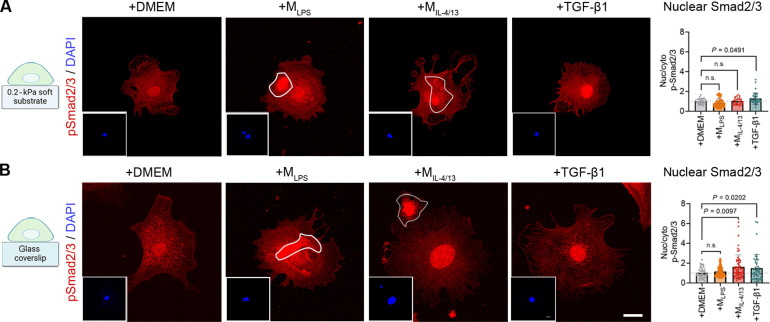
Acute Mϕ contact induces fibroblast nuclear Smad translocation on stiff substrates. Fibroblasts were grown on gelatin-coated (**A**) soft 0.2-kPa substrates or (**B**) glass coverslips for 1 hour before being immunostained for pSmad2/3 (red) and DAPI (blue, inset). Compared are fibroblasts in control medium (+DMEM), in contact with M_LPS_ or M_IL-4/13_, and treated with the Smad signaling agonist TGF-β1. Scale bar, 20 μm. The ratios of pSmad2/3 nuclear to cytoplasmic staining intensity were quantified from immunofluorescence images. Experiments were performed with cells cultured from three biological repeats, every data point representing each cell. Statistical significance was calculated from the mean values of the biological repeats (*n* = 3) using repeated measures ANOVA and Fisher’s LSD post hoc analysis with significance reached with *P* < 0.05 and “n.s.” indicating not significant.

Several days of direct contact with M_IL-4/13_ activate fibroblasts to express α-SMA in a TGF-β1–dependent manner (*9*). To test whether short contact with Mϕ also induces a canonical TGF-β1 signaling response in fibroblasts, we measured nuclear translocation of phosphorylated Smad2/3 (pSmad2/3), analogous to YAP/TAZ (Fig. 2). Unexpectedly, M_IL-4/13_ did not induce significant increases in nuclear Smad2/3 in fibroblasts spread on 0.2-kPa soft substrates within 2 hours of contact (Fig. 2A). In contrast, performing the same experiment with fibroblasts grown on stiff glass substrates resulted in robust Smad2/3 nuclear translocation upon 2 hours of contact with M_IL-4/13_ (Fig. 2B). Concomitantly, TGF-β1–induced Smad2/3 nuclear translocation was lower on soft than on stiff-grown fibroblasts (Fig. 2, A and B). Contact with M_LPS_ did not induce a Smad2/3 response in fibroblasts grown on either soft or stiff substrates (Fig. 2, A and B). These data show that acute contact with M_IL-4/13_ can elicit myofibroblast activation through YAP/TAZ but not Smad2/3 signaling in a soft environment. Next, we pursued the observation that contact with M_IL-4/13_ initiates fibroblast YAP/TAZ signaling in mechanical conditions that typically prevent myofibroblast activation.

### Fibroblast contraction is triggered by acute contact with profibrotic Mϕ

**Fig. 3.**
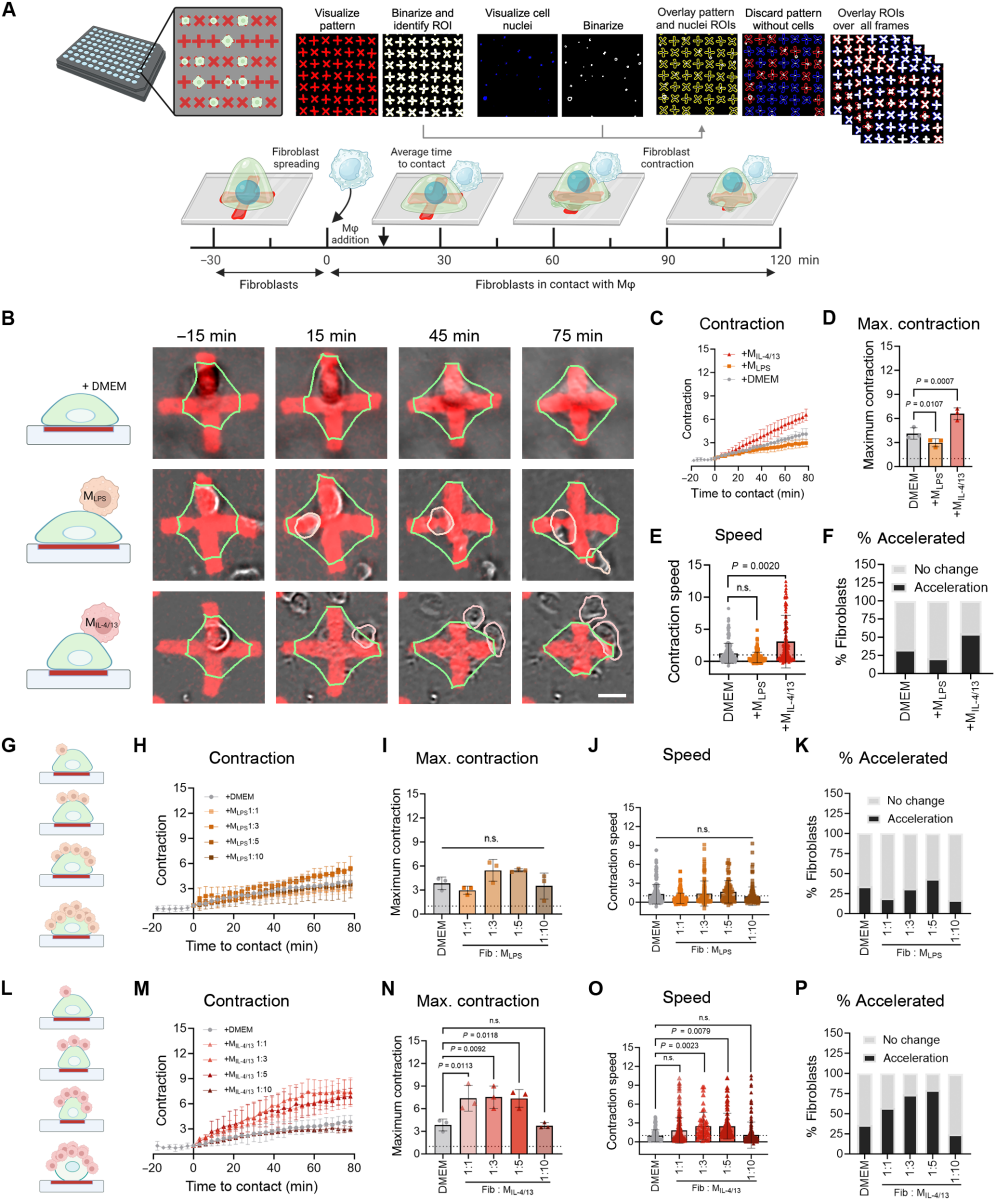
Mϕ contact enhances fibroblast contraction amplitude and speed. (**A**) Regions of interest (ROIs) of cross-shaped patterns on FLECS and nuclei were binarized and overlaid to cross patterns without cells (blue ROIs). The area fraction of the crosses was quantified in each image frame and compared to the initial frame. Fibroblasts were seeded on FLECS for 30 min before imaging to allow for cell adhesion. Mϕ were added after an additional 30 min and were imaged for a total of 2 hours. (**B**) Fibroblasts (green outlines) on FLECS pattern (red) alone or in coculture with M_LPS_ (orange outlines) or M_IL-4/13_ (pink outlines) at selected time points. Scale bar, 20 μm. (**C**) Fibroblast contraction is plotted as the inverse of the pattern area fraction over time. Time *t* = 0 is the addition of control DMEM or Mϕ contact. The means of 40 to 60 fibroblasts per individual experiment from three biological repeats are shown: (**D**) maximum contraction after 90 min and (**E**) the slope of contraction curves normalized to contraction speed before *t* = 0. (**F**) The resulting contraction speeds are either classified as accelerated compared to fibroblasts alone or not changed and plotted as a percentage. (**G**) Schematic of concentration series of M_LPS_ addition, increasing the fibroblasts: Mϕ ratio from 1:1 to 1:10. (**H** to **K**) Fibroblast contraction of patterns is analyzed as described for (C) to (F) from 20 to 60 fibroblasts per individual experiments from three biological repeats. (**L**) Schematic of concentration series of M_IL-4/13_ addition, increasing the fibroblasts: Mϕ ratio from 1:1 to 1:10. (**M** to **P**) Fibroblast contraction of patterns are analyzed as described for (C) to (F) from 20 to 60 fibroblasts per individual experiment from three biological repeats. Statistical significance was calculated using repeated measures ANOVA and Fisher’s LSD post hoc analysis with significance reached with *P* < 0.05 and “n.s.” indicating not significant.

The TAZ-citrine nuclear translocation in live fibroblasts upon M_IL-4/13_ contact was accompanied by enhanced deformation of elastic silicone culture substrates produced to wrinkle under fibroblast force (*33*) (Fig. 1C). ECM contraction is a prime function of activated fibroblasts and is causal for the stiffening of scar tissue [reviewed in (*3*)]. Thus, we propose that contact with M_IL-4/13_ can initiate the positive feedback loop between stiff ECM and myofibroblast activation that is characteristic for the development of tissue fibrosis. To quantify fibroblast contraction, we used high-throughput arrays of fluorescently labeled elastomeric contractible surfaces (FLECSs) (*34*), where fibroblast contraction is proportional to the deformation of cross-shaped adhesive micropatterns (Fig. 3A). Single fibroblasts spread over the adhesive cross patterns within 30 min after seeding and started to spontaneously and gradually reduce the area of the cross shape over a 90-min observation time (Fig. 3, B and C). Mϕ that were then added formed contacts with fibroblasts within less than 15 min on average. M_IL-4/13_ contact resulted in 1.59-fold higher maximum contraction after 90 min (Fig. 3D), and a 3-fold higher average contraction speed in 50% of the touched fibroblasts compared to controls (Fig. 3E). Only 25% of the fibroblasts accelerated their contraction above the average contraction speed in response to added DMEM control medium (Fig. 3F). Contact with M_LPS_ had no significant effect on fibroblast contraction amplitude (Fig. 3, B and C) and speed (Fig. 3E), and even reduced the percentages of fibroblasts with accelerated contraction to 10% (Fig. 3F).

The inspection of hundreds of live videos showed that Mϕ remained associated with the fibroblast over the whole observation period once the initial physical contact was established. Because generally lower numbers of M_LPS_ were found to attach to fibroblasts than M_IL-4/13_ at any given time point, we assessed whether fibroblast contraction depended on contact event frequency rather than the Mϕ type. However, despite increasing the numbers of M_LPS_ added per fibroblast from 1 to 10 (Fig. 3G), fibroblast maximum contraction (Fig. 3, H and I), contraction speed (Fig. 3J), and percentages of fibroblasts with accelerated contraction (Fig. 3K) all remained unchanged. In contrast, increasing the numbers of M_IL-4/13_ from 1 to 5 significantly enhanced all fibroblast contraction parameters (Fig. 3, M to P). Even higher M_IL-4/13_ numbers (10:1) reduced fibroblast contraction values, visibly by inducing partial or full detachment from the cross-shaped patterns because of excessive contraction (Fig. 3, L to P). Collectively, these results show that contact with M_IL-4/13_ but not M_LPS_ triggers fibroblast contractions. Higher numbers of M_IL-4/13_ enhance fibroblast contraction speed and amplitude.

**Fig. 4.**
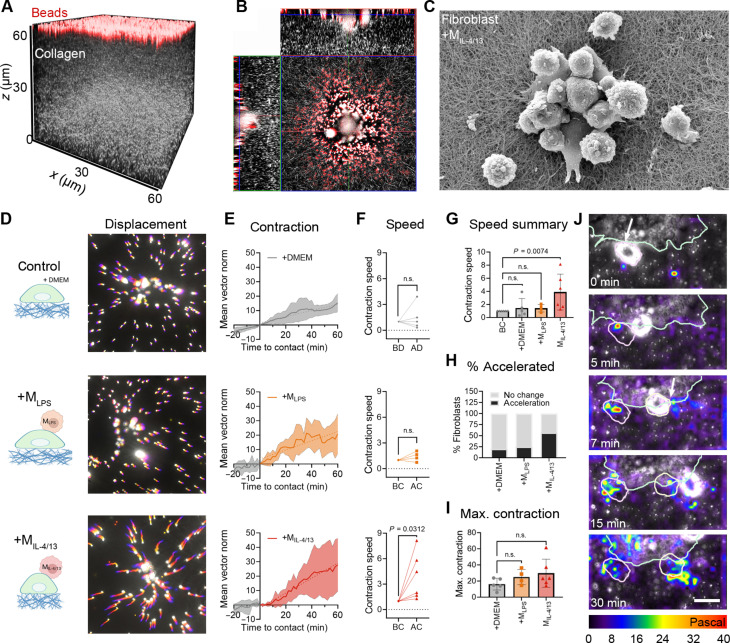
Acute profibrotic Mϕ contact induces local fibroblast contractions. (**A**) 3D confocal-reflection image of a 60-μm-thick collagen gel (white) with red fluorescent beads on the surface. (**B**) Orthogonal view of collagen gel with fibroblast (center, gray) and Mϕ (small white) in plane with fluorescent beads on the surface. (**C**) Scanning electron microscope image of fibroblast with M_IL-4/13_ on collagen gel. (**D**) To visualize bead displacement, every frame was assigned a different color and overlaid to create a temporal color-code image of fibroblasts alone, fibroblasts with M_LPS_, and fibroblasts with M_IL-4/13_ over 80 min. (**E**) Fibroblast contractions were quantified by summing the vector norms of all bead displacements (in micrometers). Averages are shown ± SD for two to three fibroblasts from three independent experiments. (**F**) Speed of fibroblast contractions measured as the slope of the curves in (E), normalized to unstimulated contraction before *t* = 0. BD, before DMEM; AD, after DMEM; BC, before contact; AC, after contact. (**G**) Summary of contraction speeds. (**H**) The resulting contraction speeds are either classified as accelerated compared to fibroblasts alone or not changed and plotted as a percentage. (**I**) Maximum contraction for each fibroblast from curves in (E); each dot represents one fibroblast. (**J**) Traction force microscopy was performed to monitor local fibroblast contractions upon Mϕ contact, with local stress (pascals) shown color coded. M_IL-4/13_ (outlined in pink) contact the fibroblast (green outline) at various contact time points; outlines of the M_IL-4/13_ remain throughout all images when contact was made in the previous frame, with arrows indicating the point of contact. Scale bar, 20 μm. Fibroblast contraction analyzed as described for (E) to (G) from four to six fibroblasts from three biological repeats. Statistical significance was calculated using repeated measures ANOVA and Fisher’s LSD post hoc analysis with significance reached with *P* < 0.05 and “n.s.” indicating not significant.

Fibroblasts deformed FLECS patterns predominantly in the region where they were contacted by M_IL-4/13_, indicating a local contractile response (Fig. 3B). To resolve such subcellular contractile events, we next seeded fibroblasts on top of 70- to 80-μm-thick, 0.2-kPa soft three-dimensional (3D) collagen gels for 60 min (*t* = 0), as done previously (*17*) (Fig. 4, A and B). Fluorescent microbeads were covalently linked to the gel surface to track in-plane collagen displacements by local fibroblast contractions (Fig. 4, A and B). Mϕ were then added (Fig. 4C), and bead displacements were recorded for another 60 min every 2 min (Fig. 4D and movie S2). Overlaying the bead positions with a different rainbow color for every acquisition time frame illustrated higher fibroblast contraction following contact with M_IL-4/13_ compared to contact with M_LPS_ or fibroblasts alone (Fig. 4D, longer “comet tails”). Bead displacements were then transformed into vector maps using particle image velocimetry (PIV) to calculate the average amplitude of induced collagen deformation per fibroblast over 60 min of Mϕ contact (Fig. 4E, “mean vector norm”). M_IL-4/13_ contact increased fibroblast contraction speeds by 3.9-fold (Fig. 4, E to G), the percentage of contraction-accelerated fibroblasts from 10 to 57% (Fig. 4H), and maximum contraction after 60 min by 1.83-fold (Fig. 4I), compared to fibroblasts alone or in contact with M_LPS_. These data established that fibroblast contractile responses to Mϕ contacts was comparable between 2D-engineered FLECS hydrogels and on top of 3D collagen gels.

### Acute profibrotic Mϕ contact induces local fibroblast membrane stress

**Fig. 5.**
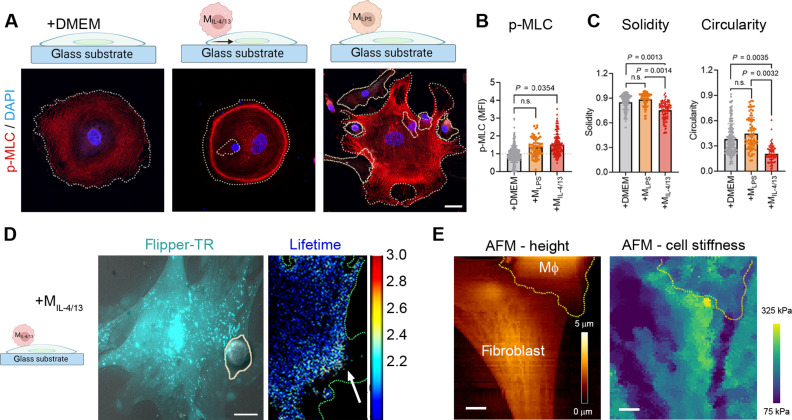
Acute profibrotic Mϕ contact induces local fibroblast stress. (**A**) Fibroblasts were spread on glass coverslips for 60 min to a circular shape before M_IL-4/13_ or medium (+DMEM) were added for another 60 min, followed by staining for p-MLC (red) and DAPI (blue). (**B**) MFI of p-MLC of fibroblasts; quantified were 90 to 100 fibroblasts in total from three biological repeats. (**C**) Deviation from regular circular fibroblast spreading was quantified using cell circularity and solidity from binary images. Circularity [4×pi×area/(perimeter)^2^] is 1 for a circle; solidity [area/convex area] is a measure of cell perimeter smoothness with 1 being perfectly smooth. (**D**) Representative image of a Flipper-TR–stained fibroblast in contact (arrow) with an unstained M_IL-4/13_ where the lifetime (nanoseconds) of the Flipper-TR probe is color coded. (**E**) AFM height image of a fibroblast in contact with a M_IL-4/13_ (yellow outline) on a stiff substrate; the force curve analysis display of Young’s moduli (kilopascals) is color coded. All scale bars, 20 μm. Experiments were performed with cells cultured from three biological repeats, where every data point represents each cell. Statistical significance was calculated using repeated measures paired *t* tests, repeated measures ANOVA, and Fisher’s LSD post hoc analysis with significance reached with *P* < 0.05 and “ns” indicating not significant.

Conversion of PIV into traction force micrographs (*35*) demonstrated a clear increase in highly localized fibroblast contraction stresses of 8 to 40 Pa within 5 min of M_IL-4/13_ contact (Fig. 4J). These local contractions lasted for at least 30 min (Fig. 4J, outlined by the first Mϕ contact position over time), even after the Mϕ moved on to contact another fibroblast area (Fig. 4J, arrows). As another means to quantify how M_IL-4/13_ induce local fibroblast contractions, we seeded fibroblasts onto gelatin-coated stiff glass surfaces for 60 min after which they spread to a near-perfect circular cell shape (Fig. 5A). M_IL-4/13_ contact enhanced the average staining intensity of phosphorylated myosin light chain (p-MLC) in spreading fibroblasts by 1.4-fold, which is a measure for myosin activation (Fig. 5B). To visualize the local effects of Mϕ contact, we exploited that application of local mechanical stress breaks circular symmetry by inducing local retractions and thereby results in fibroblast shape polarization on nondeformable (glass) substrates (*36*). Adding M_IL-4/13_ caused local membrane retractions wherever fibroblasts were touched by M_IL-4/13_ (Fig. 5A), resulting in a 1.68-fold and 1.14-fold decrease in “solidity” and circularity of the fibroblast circumference, respectively (Fig. 5C). Solidity is calculated as cell area divided by the area of its convex hull that outlines the cell shape like a rubber band. As a measure for the degree of concavity, solidity is maximal (1) when a cell has no irregular borders and cavities. No changes were observed in fibroblast p-MLC intensity, circularity, or solidity upon contact with M_LPS_ (Fig. 5, A to C).

Because p-MLC staining intensity was highest where M_IL-4/13_ touched, we postulated higher fibroblast tension in the contact areas. To directly assess fibroblast membrane tension upon M_IL-4/13_ contact, we first performed fluorescent lifetime imaging microscopy (FLIM). FLIM demonstrated higher fluorescent lifetimes of the membrane tension–sensitive dye Flipper-TR, and thus higher membrane tension, in the contact area between fibroblast and M_IL-4/13_ (Fig. 5D). Probing local stiffness distributions between fibroblast-M_IL-4/13_ with atomic force microscopy (AFM) revealed relatively higher fibroblast stiffness in the contact region (Fig. 5E), consistent with the accumulation of p-MLC (Fig. 5B). Collectively, these results demonstrate that contact with M_IL-4/13_ but not M_LPS_ induces fibroblast contractions and mechanical stress that originate at the contact site.

### Acute M_IL-4/13_ contact induces Ca^2+^ influx and NFAT1 nuclear translocation in fibroblasts

**Fig. 6.**
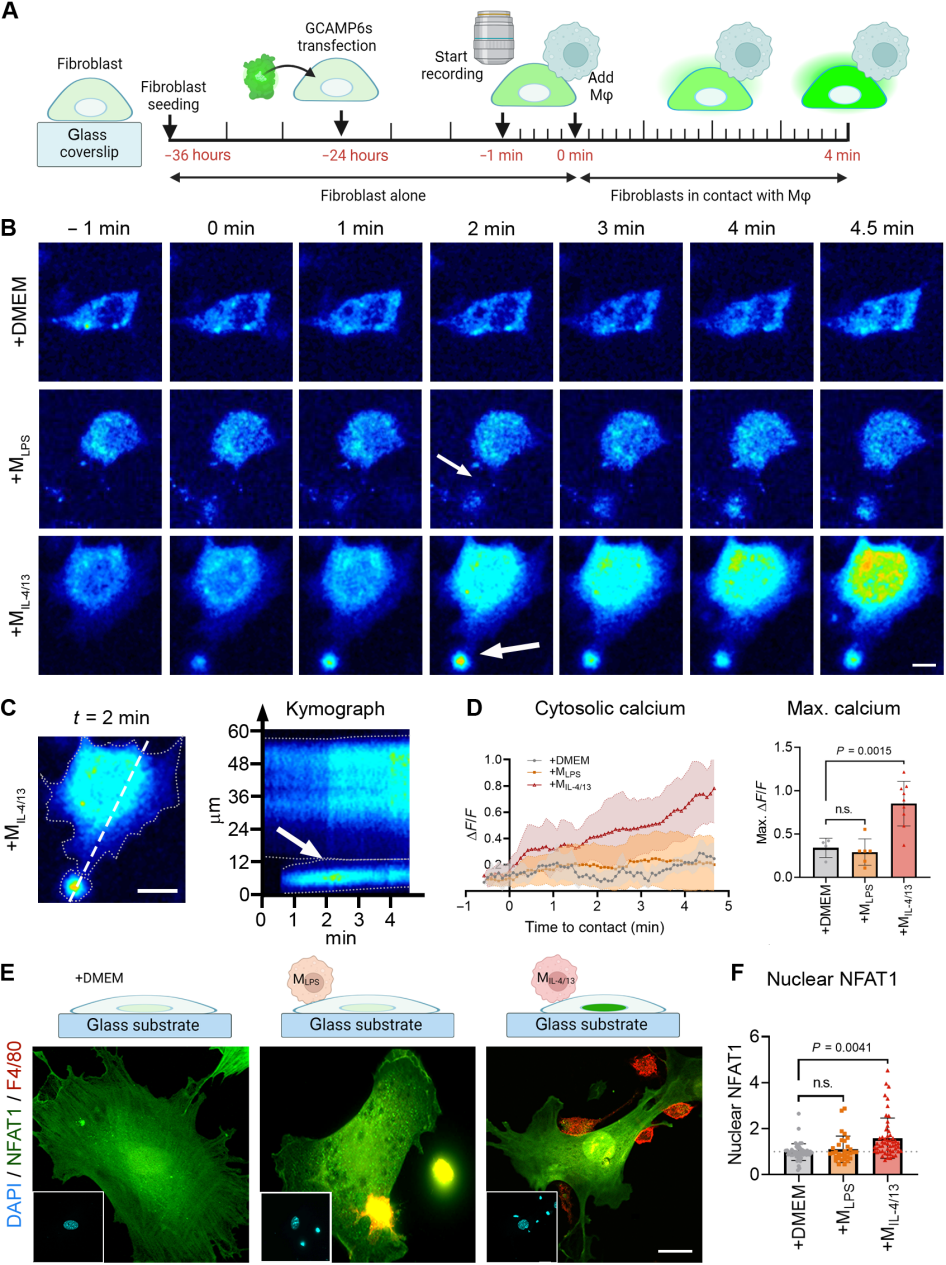
Acute profibrotic Mϕ induced calcium influx in fibroblasts. (**A**) Fibroblasts were seeded onto gelatine-coated glass coverslips for 12 hours before being transfected with GCaMP6s, which fluoresces upon binding of Ca^2+^ and imaged after another 24 hours. After 1 min of imaging, M_LPS_ or M_IL-4/13_ was added. (**B**) Ca^2+^ imaging (1 frame/5 s) of a fibroblast-Mϕ contact (arrow) scenario. Scale bar, 10 μm. (**C**) Kymograph over the fibroblast- M_IL-4/13_ contact region (dashed line) where the white arrow points to the contact moment. Scale bar, 10 μm. (**D**) GCaMP6s fluorescence intensity of the fibroblast was quantified and normalized to fluorescence at the beginning of the recording, and mean values were plotted with SD over time. The bar graph summarizes the maximum Ca^2+^ intensities of one to three fibroblasts from three experiments. (**E**) To follow the downstream effects of Ca^2+^ influx, fibroblasts were stained for NFAT1 (green) and F4/80 (red) to distinguish between fibroblast and Mϕ. DAPI (blue, insets). Scale bar, 20 μm. (**F**) Quantification of nuclear levels of NFAT1 of 10 to 20 fibroblasts from three biological repeats shown as averages with SD. Statistical significance was calculated using repeated measures ANOVA and Fisher’s LSD post hoc analysis with significance reached with *P* < 0.05 and “ns” indicating not significant.

Phosphorylation of MLC is regulated by the MLC kinase (MLCK), allowing myosin to bind to actin filament within stress fibers (*37*). Because MLCK activity in fibroblastic cells is regulated by increases in the concentration of cytosolic Ca^2+^ via binding of calmodulin (*38*), we tested the involvement of Ca^2+^ in M_IL-4/13_-induced fibroblast contraction. Fibroblasts grown on gelatin-coated glass coverslips were transfected with GCaMP6s, which fluoresces upon binding of Ca^2+^ due to a conformational change of its calmodulin moiety (Fig. 6A). Contact with M_IL-4/13_ resulted in an increase in GCaMP6s fluorescence within 5 min (Fig. 6B), through a wave of Ca^2+^ starting in the fibroblast at the M_IL-4/13_ contact site (Fig. 6C, arrow, and movie S3), as resolved using kymograph analysis (*39*). Cytosolic Ca^2+^–dependent GCaMP6s fluorescence was significantly higher (1.6-fold) already 1 min after M_IL-4/13_ contact and continued to increase up to 3.59-fold compared to control (Fig. 6D). As another indicator of active Ca^2+^, we assessed the nuclear factor of activated T cells 1 (NFAT1). NFAT1 translocates to the nucleus upon Ca^2+^-dependent activation of calcineurin with kinetics comparable to those of Smad2/3 and YAP/TAZ. Fibroblasts in contact with M_IL-4/13_ had 1.7-fold higher nuclear NFAT1 levels than untouched fibroblasts (Fig. 6, E and F). No significant changes in cytosolic Ca^2+^–dependent GCaMP6s fluorescence or nuclear NFAT1 were observed in fibroblasts after contact with M_LPS_ (Fig. 6, B to F). Thus, contact with M_IL-4/13_ but not M_LPS_ results in an increase of cytosolic Ca^2+^ that propagates from the contact site and is followed by downstream nuclear translocation of NFAT1 in fibroblasts.

### Piezo1 mediates Ca^2+^ influx and NFAT1 nuclear translocation in fibroblasts upon Mϕ contact

**Fig. 7.**
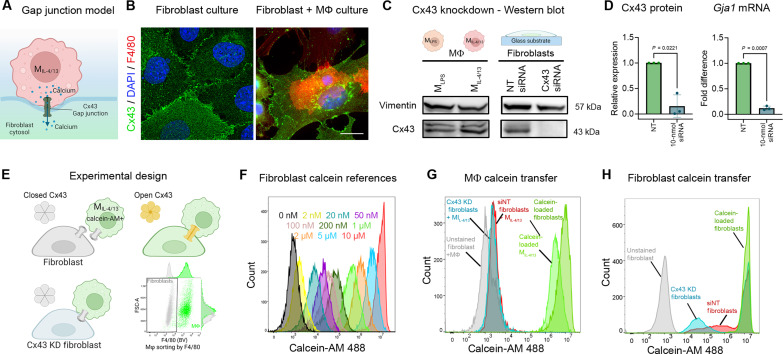
Cx43 does not mediate direct communication between fibroblasts and M_IL-4/13_. (**A**) Working model of Cx43 formation between Mϕ and fibroblasts. (**B**) Representative image of Cx43 expression between fibroblast-fibroblasts and fibroblast-Mϕ. Cx43 (green), DAPI (blue), and F4/80 (red). Scale bar, 20 μm. (**C**) Protein level of Cx43 in Mϕ treated with LPS (M_LPS_) or IL-4/13 (M_IL-4/13_), and fibroblasts with nontargeting (NT) siRNA or 10 nmol of Cx43 siRNA. (**D**) Quantification of relative protein and mRNA expression of Cx43 after knockdown. (**E**) Schematic of gap junction assay. (**F**) Standard curve of increasing calcein-AM concentrations in fibroblasts. Fibroblasts loaded with calcein-AM from 0 to 10 μm. (**G**) Flow histograms of unstained fibroblasts and M_IL-4/13_ coculture (gray), wild-type (WT) fibroblasts (red) in coculture with 10 μM calcein-AM loaded M_IL-4/13_, Cx43 knockdown fibroblasts (blue) in coculture with 10 μM calcein-AM loaded M_IL-4/13_, and 10 μM calcein-AM in fibroblast and M_IL-4/13_ monocultures (green); F4/80-positive cells were sorted out to differentiate between fibroblasts and M_IL-4/13_. (**H**) Flow histograms of unstained fibroblasts (gray), WT fibroblasts (red), Cx43 knockdown fibroblasts (blue), 10 μM calcein-AM in fibroblast monoculture (green). Statistical significance was calculated using paired *t* tests with significance reached with *P* < 0.05.

To address how M_IL-4/13_ trigger the Ca^2+^ response in fibroblasts, we tested two possible scenarios dependent on physical contact: (i) direct transmission of Ca^2+^ from Mϕ to fibroblasts through heterocellular gap junctions and (ii) opening of SACs in the fibroblast membrane by Mϕ touch. Of the gap junction proteins, connexin-43 (Cx43) is expressed in both fibroblasts and Mϕ and regulates their activation during tissue repair and fibrosis (*40*, *41*). Concomitantly, Cx43 was expressed and localized to homocellular junctions in cultured lung mouse fibroblasts as we showed previously for rat fibroblasts (*41*) (Fig. 7, A and B). Although M_IL-4/13_ and M_LPS_ expressed comparable levels of Cx43, the protein did not localize to Mϕ-fibroblast junctions (Fig. 7C, shown for M_IL-4/13_). To functionally test whether Cx43 gap junctions mediate Ca^2+^ transfer from M_IL-4/13_, we first knocked down Cx43 in fibroblasts with a smart pool of four targeting short interfering RNAs (siRNAs), resulting in ~90% reduction in Cx43 protein and mRNA (*Gja1*) expression (Fig. 7, C and D). Next, we set up contact pairs of fibroblasts with M_IL-4/13_ that have been preloaded with 10 μM calcein-acetoxymethyl (AM) and later gated out as F4/80-positive M_IL-4/13_ in flow cytometry (Fig. 7E); calcein-AM is a green, fluorescent dye small enough to pass through functional gap junctions. For reference, fibroblasts were directly loaded with calcein-AM in a dilution series (Fig. 7F). The calcein-AM concentration in the recipient fibroblasts after 2 hours of coculture was estimated to be only 2 nM, as extrapolated from the reference (Fig. 7, F to H). Knockdown of Cx43 in fibroblasts did not further reduce the low levels of calcein-AM transfer from the preloaded M_IL-4/13_ but eliminated fibroblast-fibroblast calcein exchange in control experiments (Fig. 7, G and H). Thus, although both fibroblasts and M_IL-4/13_ express Cx43, they do not seem to form functional gap junctions to mediate direct exchange of small molecules like calcein-AM or Ca^2+^.

**Fig. 8.**
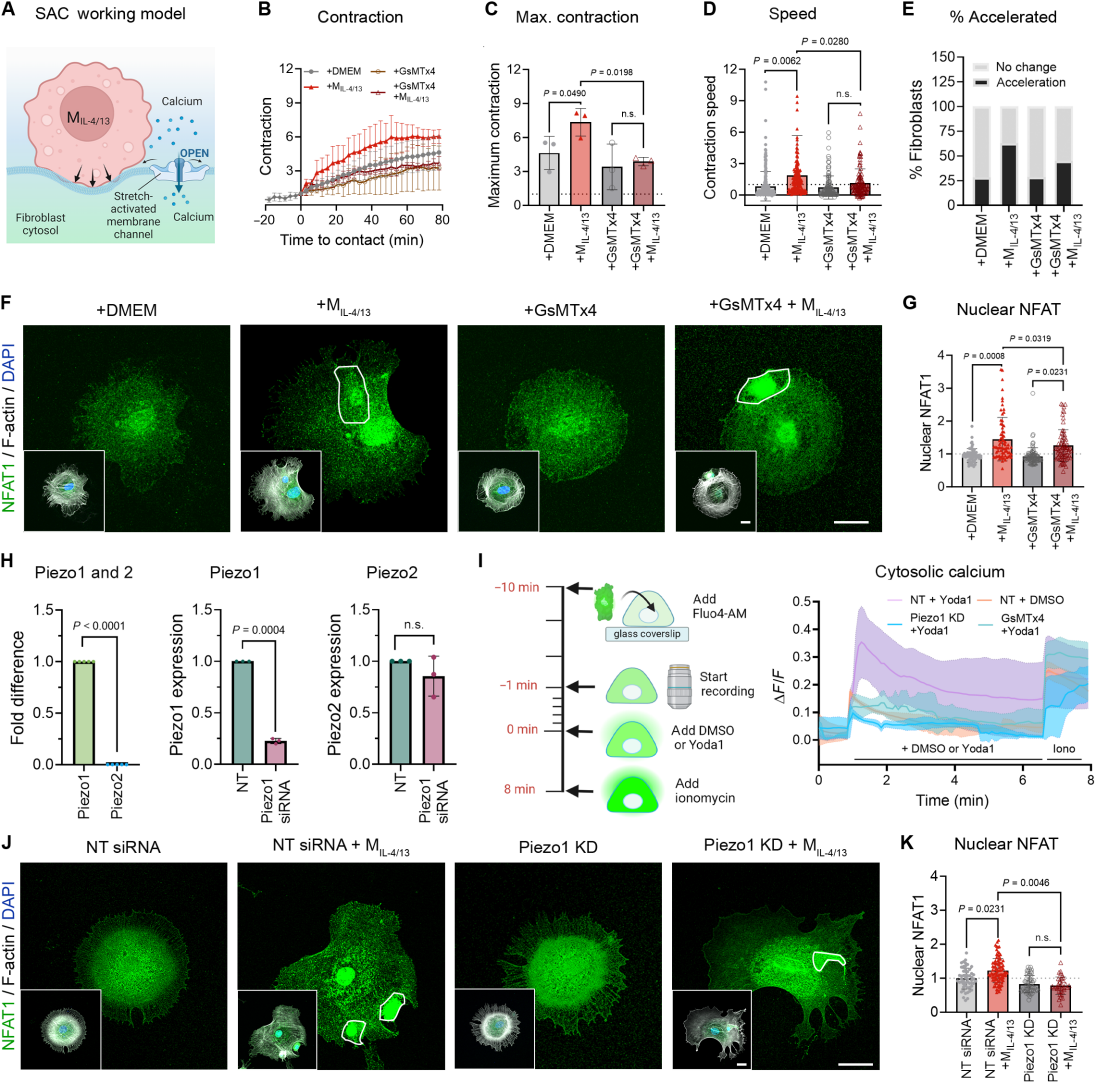
Piezo1 mediates cytosolic Ca^2+^ elevation in fibroblasts upon Mϕ contact. (**A**) SAC working model. (**B**) Fibroblast contraction of FLECS patterns is shown after pretreatment with GsMTx4 with time *t* = 0 indicating the addition of control DMEM or Mϕ contact. Mean values from 50 to 80 fibroblasts per experiment are shown for three biological repeats: (**C**) maximum contraction after 90 min and (**D**) contraction speed. (**E**) Percentages of fibroblasts with accelerated contraction over to fibroblasts alone are shown as percentage of all fibroblasts. (**F**) The Ca^2+^ downstream effector NFAT1 (green) was costained with DAPI (blue, inset) and F-actin (gray, inset) in control fibroblasts (+DMEM), pretreated with SAC inhibitor GsMTx4, with and without contacting M_IL-4/13_ (outlined). (**G**) Nuclear NFAT1 immunofluorescence staining intensity was quantified over 20 to 30 fibroblasts per experiment; shown are averages (±SD) from four experiments. (**H**) Fibroblasts mRNA levels of Piezo2 and Piezo1 are shown in comparison and after Piezo1 knockdown as averages (±SD) from three to five experiments. (**I**) Cytosolic Ca^2+^ levels were measured using Fluo-4 AM in Piezo1 knockdown fibroblasts and fibroblasts treated with dimethyl sulfoxide (DMSO) (control) or Yoda1 (Piezo1 agonist). After 8 min, fibroblasts were treated with ionomycin to open all Ca^2+^ stores. (**J**) Fibroblasts transfected with nontargeting (NT) or Piezo1 siRNA, kept in control medium (+DMEM) or contacted with M_IL-4/13_ for 2 hours before being stained for NFAT1 (green), DAPI (blue), and F-actin (gray). (**K**) Nuclear NFAT1 staining intensity is quantified from images of 20 to 40 fibroblasts per experiment; shown are averages (±SD) from three biological repeats. All scale bars, 20 μm. Statistical significance was calculated using repeated measures paired *t* tests, repeated measures ANOVA, and Fisher’s LSD post hoc analysis with significance reached with *P* < 0.05 and “ns” indicating not significant.

With a gap junction transfer being ruled out as a main contact-mediated mechanism, we proceeded to test the involvement of fibroblast SACs as mediators of M_IL-4/13_-induced cytosolic Ca^2+^ elevation and subsequent contraction of fibroblasts (Fig. 8A). Addition of the generic SAC inhibitor GsMTx4 to fibroblasts in FLECS contraction tests completely abolished M_IL-4/13_-induced increases in fibroblast contraction amplitude, speed, and percentages of fibroblasts with accelerated contraction obtained without the drug (Fig. 8, B to E). Concomitantly, SAC inhibition with GsMTx4 prevented the Ca^2+^-dependent nuclear translocation of NFAT1 in fibroblast upon contact with M_IL-4/13_ (Fig. 8, F and G). Thus, SACs are required for M_IL-4/13_ induction of fibroblast contraction via cytosolic Ca^2+^ elevation. To identify the fibroblast SAC responsible for sensing M_IL-4/13_ contacts, we focused on the Piezo family of mechanically activated cation channels. Piezo channels play important roles in detecting ECM elasticity and regulating mechanically mediated fibroblast behavior in lung fibrosis (*42*, *43*). Of the two known Piezo channels, we found Piezo1 to be expressed in our primary mouse lung fibroblasts at 2000-fold higher levels than Piezo2 (Fig. 8H). Knocking down Piezo1 with a smart pool of four targeting siRNAs reduced the levels of Piezo1 mRNA to 23% without compensation by Piezo2 (Fig. 8H). In functional tests, the Piezo1 agonist Yoda1 stimulated a Ca^2+^ response in fibroblasts transfected with nontargeting siRNA, whereas Piezo1-deficient fibroblasts and fibroblasts treated with GsMTx4 were nonresponsive to Yoda1 (Fig. 8I). Consistently, knockdown of Piezo1 in fibroblasts prevented Ca^2+^-dependent nuclear translocation of NFAT1 upon contact with M_IL-4/13_ (Fig. 8, J and K). Collectively, our results show that Piezo1 mediates the M_IL-4/13_-induced cytosolic Ca^2+^ increases that drive subsequent fibroblast contractions.

### The Mϕ_IL-4/13_ integrin αvβ3 is required to elicit Ca^2+^ signaling and contraction in fibroblasts

**Fig. 9.**
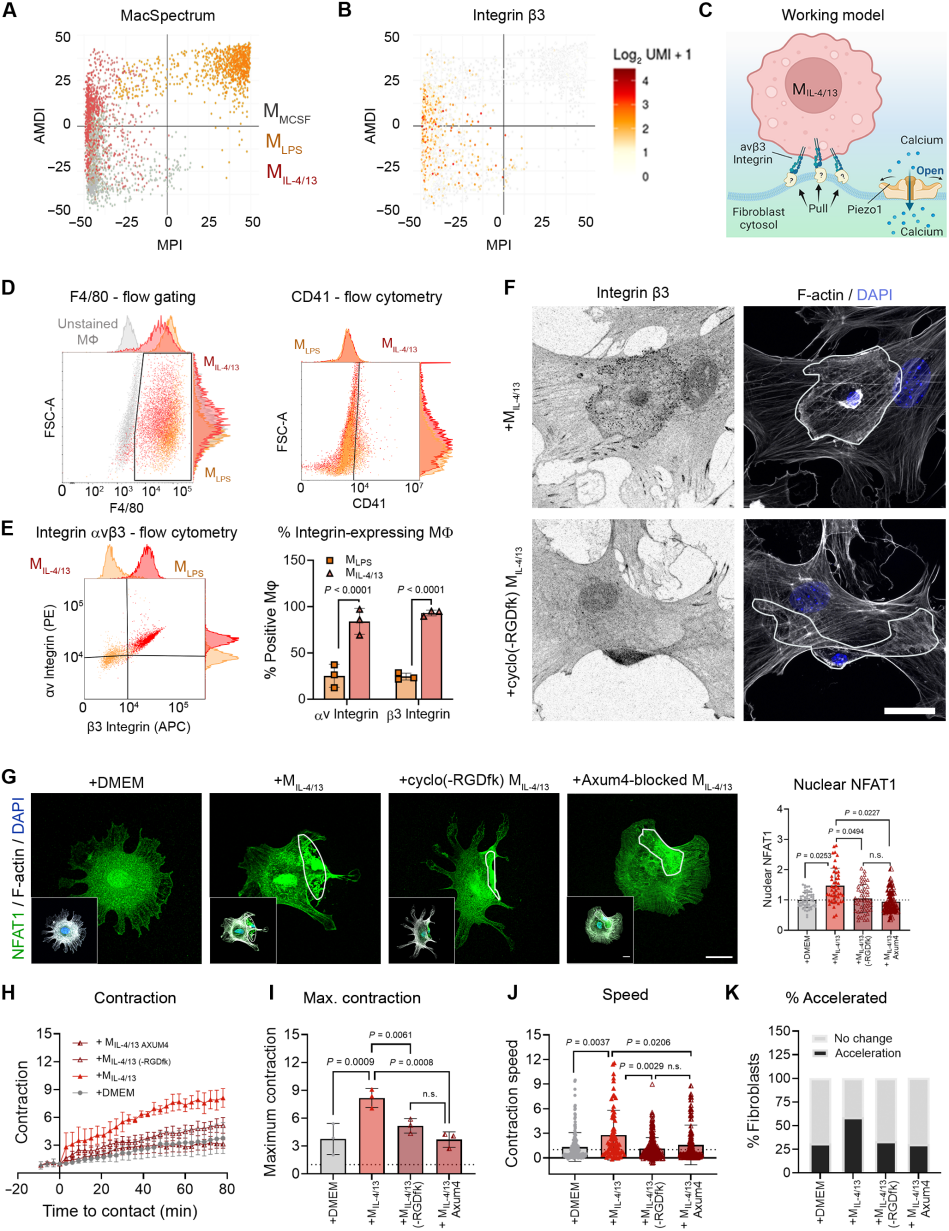
The Mϕ integrin αvβ3 mediates fibroblast Ca^2+^ and contraction responses. (**A**) Single-cell datasets of naïve (M-CSF only), LPS, or IL-4/13–treated Mϕ from Li and co-workers. The MacSpectrum situates Mϕ polarization states by activation-induced Mϕ differentiation index (AMDI)—the degree of Mϕ terminal maturation, versus Mϕ polarization index (MPI)—the polarity of Mϕ activation. (**B**) Using the author’s MacBrowser, M_IL-4/13_ were found to uniquely express β3 integrin. (**C**) Working model of M_IL-4/13_ αvβ3 integrin pulling on the fibroblast membrane. (**D**) Mϕ were stained for αv and β3 integrin and analyzed by flow cytometry. F4/80-positive M_LPS_ (orange) and M_IL-4/13_ (red) were compared to unstained Mϕ (gray) and gated to confirm the absence of the platelet-specific αIIb (CD41) and expression of (**E**) αv and β3 integrin. (**F**) M_IL-4/13_ were pretreated with αvβ3 integrin inhibitor cyclo(-RGDfk) or control and spread on fibroblasts before being stained after 1 hour for β3 integrin (black, inverted immunofluorescence), F-actin (gray), and DAPI (blue); Mϕ are outlined. (**G**) Fibroblast Ca^2+^ responses were assessed by staining for NFAT1 (green), DAPI (blue, inset), and F-actin (gray, inset). Nuclear NFAT1 was quantified from 20 to 30 fibroblasts per three biological experiments. (**H**) Fibroblast contraction of FLECS patterns was quantified in control medium (+DMEM) or with M_IL-4/13_, αvβ3 integrin–blocked M_IL-4/13_, or anti–β3 integrin antibody (Axum4)–blocked M_IL-4/13_. Time *t* = 0, the addition of control DMEM or Mϕ contact. (**I**) Maximum contraction after 90 min and (**J**) the slope of contraction curves normalized to contraction speed before *t* = 0. (**K**) Percentages of fibroblasts with accelerated contraction over to fibroblasts alone are shown as percent of all fibroblasts. Shown are the averages of 30 to 50 fibroblasts per experiment from three biological repeats. Scale bars, 20 μm. Statistical significance was calculated using repeated measures ANOVA and Fisher’s LSD post hoc analysis with significance reached with *P* < 0.05 and “ns” indicating not significant.

While acute contact with M_IL-4/13_ enhanced fibroblast contraction, Ca^2+^, and YAP signaling, contact with M_LPS_ did not. Thus, we searched for unique molecules on the surface of M_IL-4/13_ that potentially mediate strain in the fibroblast membrane to open SACs. We first screened for Mϕ adhesion receptors using publicly available single-cell RNA sequencing data, produced from mouse bone marrow–derived Mϕ populations polarized identically to our conditions (Fig. 9A, “MacSpectrum”) (*10*). Among the signature gene list of adhesion proteins, the integrin β3 subunit (CD61) was expressed uniquely in M_IL-4/13_ and near absent in M_LPS_ (Fig. 9B). Thus, we pursued whether integrin β3 mediates M_IL-4/13_ contact-induced stress in fibroblasts (Fig. 9C). The two possible α integrin partners of β3 integrin are αv integrin (CD51) and αIIb integrin (CD41), of which the latter is exclusively expressed in platelets (*44*). Consistently, following gating for F4/80-positive Mϕ, CD41 was not detected on M_LPS_ and M_IL-4/13_ in flow cytometry (Fig. 9D). Both αv and β3 integrins were expressed on the surface of most M_IL-4/13_ (84 and 93%, respectively) but only in a small fraction of M_LPS_ (25 and 24%, respectively) (Fig. 9E).

**Fig. 10.**
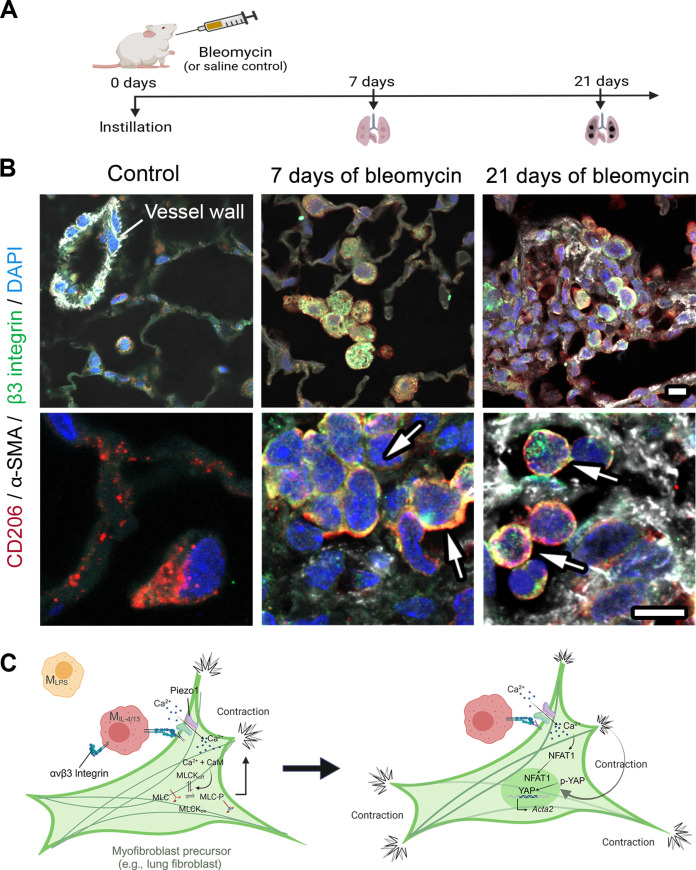
CD206 and αvβ3 integrin–positive Mϕ characterize induced mouse lung fibrosis. (**A**) Mouse model of bleomycin-induced lung fibrosis; controls are mouse lungs excised 21 days after saline injection. (**B**) Paraffin tissue sections were produced from mouse lungs 7 and 21 days after the instillation of bleomycin or saline (21 days) as control. Immunofluorescence shows expression of β3 integrin (green), the profibrotic Mϕ marker CD206 (red), the myofibroblast marker α-SMA (white), and cell nuclei (DAPI, blue) in low- (top row) and high-magnification (bottom row) confocal images. Arrows are pointing to β3 integrin and CD206 double-positive Mϕ. (**C**) Summary: A recruited Mϕ establishes contact with a myofibroblast precursor, reinforced through αvβ3 integrins. The mechanical attachment of the Mϕ contact results in increased membrane tension in the fibroblast. Increased membrane tension opens Piezo1, which introduces an influx of Ca^*2*+^ into the fibroblast. The influx of Ca^*2*+^ can result in phosphorylation of myosin light chain that would induce contraction and/or translocate NFAT1 into the nucleus. Repeated and persistent contractions alter the cytoskeleton of fibroblast and allow nuclear translocation of YAP and enhanced expression of myofibroblast-related genes such as Acta2. Continuous YAP activation eventually leads to myofibroblast activation. Created with BioRender.com.

To explore the function of αvβ3 integrin in mediating activation of fibroblasts by acute M_IL-4/13_ contact, we pretreated M_IL-4/13_ with cyclo(-RGDfK), a potent selective inhibitor of αvβ3 integrin and with the β3 integrin–blocking antibody Axum4 (*45*). Without inhibitor added, M_IL-4/13_ spread onto and formed β3 integrin–positive contact points with fibroblasts, which were absent when M_IL-4/13_ were pretreated with either of the αvβ3 integrin–blocking agents (Fig. 9F). Inhibition of αvβ3 integrin on M_IL-4/13_ using cyclo(-RGDfK) and Axum4 antibody further reduced contact-induced and Ca^2+^-dependent NFAT1 nuclear translocation (Fig. 9G) and fibroblast contraction (Fig. 9, H to K). Last, in vivo validation using a mouse model of bleomycin-induced lung fibrosis (Fig. 10A) confirmed the expression of β3 integrin in CD206-positve profibrotic Mϕ 7 and 21 days after treatment but not in alveolar Mϕ of saline-treated control lungs (Fig. 10B). In highly fibrotic lungs (21 days of bleomycin), β3 integrin and CD206 double-positive Mϕ were found in proximity of α-SMA–positive myofibroblasts (Fig. 10B). Together, these data support that αvβ3 integrin expression on M_IL-4/13_ is required to promote acute contact-mediated fibroblast activation.

## DISCUSSION

One early key function of activated fibroblasts during the repair of physically damaged tissues is to produce the fibrous collagen that restores lost ECM structure [reviewed in (*46*)]. During remodeling of initially soft ECM into mechanically resistant and stiff scar tissue, fibroblasts acquire contractile actin-myosin bundles (stress fibers), which are the defining feature of myofibroblasts [reviewed in (*26*)]. Myofibroblasts characteristic for the later stages of tissue repair and fibrotic lesions neo-express α-SMA, which confers enhanced contractile strength to stress fibers (*33*). Fibroblast-to-myofibroblast transition is critically dependent on the mechanical resistance of their substrate (i.e., ECM stiffness) even in the presence of the most potent profibrotic cytokine TGF-β1 (*47*, *48*). How fibroblast activation into highly contractile myofibroblast is initiated in the mechanically nonpermissive conditions of a soft environment is the yet unresolved “chicken-or-egg” question. Our data support that prorepair Mϕ can kick-start myofibroblast activation in a process that depends on physical Mϕ binding to the fibroblast surface via αvβ3 integrins and opening of the fibroblast SAC Piezo1. Mϕ contact triggers a Ca^2+^ rise within seconds and subsequent enhanced fibroblast contraction within few minutes, ultimately resulting in accelerated collagen remodeling (Fig. 10C). Higher cytoskeletal stress also translates into the translocation of mechanosensitive cotranscription factors YAP and TAZ within minutes to an hour to drive the expression of myofibroblast-associated genes, including *Acta2* (α-SMA). Knockdown and inhibition of Piezo1 on the fibroblast surface or αvβ3 integrin on the Mϕ surface were efficient in blocking Mϕ-induced fibroblast activation and represent promising strategies to control scar contracture and tissue stiffening.

Intriguingly, only contact with profibrotic IL-4– and IL-13–polarized Mϕ but not LPS-polarized proinflammatory Mϕ induced myofibroblast activation, likely because M_LPS_ are lacking the mechanical stress–mediating αvβ3 integrin. Thus, in principle, contact with early proinflammatory Mϕ does not activate fibroblasts into contractile myofibroblasts. Myofibroblast activation would indeed be counterproductive in the early stages of healing because it comes at the expense of fibroblast proliferation and/or migration (*49*). Instead, initiation of contractile myofibroblasts by the later prorepair and/or profibrotic Mϕ would be restricted to tissue repair stages that already advanced to the formation of collagen-rich neo-ECM. In fibrosis, the frequency of such encounters would be higher because of the accumulation of profibrotic Mϕ in fibrotic mouse lungs described in our experiments and that of others (*9*, *50*). This idea of spatiotemporally controlled fibroblast-Mϕ interactions is consistent with our earlier studies showing that prolonged contact between prorepair Mϕ and myofibroblasts creates a spatial niche of active TGF-β1 that promotes persistent expression of α-SMA (*9*). Such prolonged Mϕ-myofibroblast contacts are maintained through heterocellular cadherin-11 junctions. However, cadherin-11 is not at play in the acute contact scenario because the “naïve” fibroblasts used in our present study do not (yet) express cadherin-11 (*9*, *51*). Further, the absence of a Smad2/3 response of fibroblasts leads us to rule out a prominent role of TGF-β1 signaling in mediating the acute response to Mϕ contact, at least in a soft environment. Therefore, Ca^2+^ signaling elicited by binding of M_IL-4/13_ αvβ3 integrin is an exciting mechanism to initiate mechanical activation of fibroblasts.

While the expression of αvβ3 integrin provides contact specificity, Piezo1 SACs expressed by fibroblasts transduce the extracellular mechanical stress exerted by M_IL-4/13_ contacts into intracellular Ca^2+^ signals. Mechanical stimulation of Piezo1 has been shown previously to elicit downstream signaling cascades that drive myofibroblast activation in animal models of kidney fibrosis (*52*), heart fibrosis (*53*), and hypertrophic scarring of skin (*54*). Piezo1 has been found in focal adhesions, where it contributes to fibroblast sensing of ECM stiffness (*55*), and mediates mechanical stress responses in cultured cardiac fibroblasts (*56*). Concomitant with our findings, stimulation of Piezo1 in fibroblast-like pancreatic stellate cells enhances MLC phosphorylation and contraction in 3D ECM (*57*). Our own earlier studies demonstrated that SACs coordinate the contractile activities of small groups of myofibroblasts that are physically connected though cadherin-11 intercellular junctions (*41*). Thus, SACs are ideally positioned to adapt fibroblast contractile remodeling activities to the mechanical state of the ECM and the identity of neighboring cells by coordinating Ca^2+^ influx in close feedback loops (*58*). Although disabling Piezo1 function was sufficient to obliterate the fibroblast response to contacting M_IL-4/13_ in our experiments, SACs of different molecular composition may mediate Mϕ communication with other fibroblast types. For instance, the fibroblast SAC transient receptor potential vanilloid 4 (TRPV4) plays a role in various fibrotic conditions and is directly involved in fibroblast-to-myofibroblast activation (*59*, *60*).

In addition to Piezo1, we assessed the role of Cx43 gap junctions as another possible mode of Ca^2+^ signaling between Mϕ and fibroblasts because our earlier studies established the involvement of Cx43 in mediating Ca^2+^ signal exchanges between fibroblasts (*41*). Expression of Cx43 gap junctions is elevated in conditions of organ injury and fibrosis and shown to regulate fibroblast activation through Ca^2+^ signaling (*61*–*63*). Cx43 is also highly expressed in Mϕ in similar pathological settings (*64*, *65*) and found in both M_IL-4/13_ and M_LPS_ in our study. Given the multitude of studies demonstrating the expression of Cx43 on both Mϕ and fibroblasts, including our present work, it was unexpected that fibroblasts knocked out for Cx43 were still activated by M_IL-4/13_ contact. Furthermore, Mϕ-fibroblast heterocellular pairs did not exchange the gap junction–permeable small molecule calcein-AM, in contrast to fibroblast homocellular pairs. It is conceivable that Cx43 in Mϕ mainly promotes the release of adenosine triphosphate (ATP) through Cx43 hemichannels (*40*), rather than forming heterocellular connexons with fibroblasts. In turn, extracellular ATP can act on fibroblasts as shown in elegant in vitro models of lung fibrosis (*66*). In the latter study, Cx43 hemichannel release of ATP from Mϕ triggered Ca^2+^ responses in distant (i.e., noncontacting) cocultured fibroblasts, which was not observed in our experiments. It remains to be shown whether M_IL-4/13_ in our experiments do not release ATP or whether our cultured lung fibroblasts do not express P2rx4, which the authors identified as the responsible ATP receptor and Ca^2+^ channel (*66*).

One immediate outcome of Mϕ-induced cytosolic Ca^2+^ increases is a local contractile response that results in local membrane retractions of fibroblasts grown on stiff substrates and globally enhanced fibroblast contraction of deformable wrinkling silicone substrates, patterned hydrogel substrates, and fibrous collagen networks. These responses are consistent with our earlier findings of fibroblasts responding to local or oscillatory cytosolic Ca^2+^ increases with contractile events (*41*). As proposed earlier and shown here, these enhanced contractile events result in accelerated ECM contraction, speeding up the ECM stiffening that feeds the mechanical loop eventually promoting full myofibroblast activation [reviewed in (*67*, *68*)]. Acutely enhanced contraction is a manifestation of higher cytoskeletal stress that also drives nuclear translocation of the mechanosensitive cotranscription factors YAP and TAZ (*32*, *69*), where they control the transcription of myofibroblast- and fibrosis-associated genes, including such that promote longer-lasting contraction like α-SMA (*29*, *70*). Consequently, inhibition of YAP using verteporfin is effective in reducing myofibroblast and scar formation in experimental models of animal fibrosis (*29*, *71*). While mechanical stress–mediated YAP/TAZ nuclear translocation is well established in the fibroblast activation process, possible direct effects of SAC stimulation and elevated Ca^2+^ on YAP/TAZ signaling and fibroblast activation are less well understood. Recently published studies are starting to establish direct links between Piezo1 mechanotransduction and YAP activation in fibroblastic phenotypes driving fibrosis of the kidneys (*52*), heart (*56*), and oral submucosa (*72*). Such converging signaling effects of YAP in fibrosis, in addition to mechanical stress, have been reported for signaling through statins (*73*) and specific G protein–coupled receptors (*74*).

Whether there is a link between Mϕ contact–mediated translocation of NFAT1, which we used here as a mere indicator of prolonged Ca^2+^ signaling, and myofibroblast activation also remains to be shown. Published data show that Ca^2+^-dependent shuttling of NFAT1 into the fibroblast nucleus is involved in regulating the expression of α-SMA expression, fibronectin, and collagen (*75*, *76*). As a downstream signaling factor of the Ca^2+^ channel transient receptor potential cation channel subfamily C member 6 (TRPC6) and the phosphatase calcineurin, NFAT1 has been shown to promote myofibroblast activation of dermal and cardiac fibroblasts (*75*, *77*). Another open question raised by our study is how αvβ3 integrin expressed on M_IL-4/13_ mediates pulling on the fibroblast membrane and opening of Piezo1. In upcoming studies, we will strive to identify potential αvβ3 integrin ligands on fibroblasts, such as urokinase-type plasminogen activator and its receptor (*78*). Identifying the αvβ3 integrin ligand will add to the growing list of molecular components mediating Mϕ-fibroblast mechanotransduction, like αvβ3 integrin, Piezo1, and NFAT1. The putative ligand would likely also provide specificity to αvβ3 integrin binding over the binding of platelet αvβIIb integrin. The prospect of blocking specific Mϕ-fibroblast contact mechanisms offers a potentially transformative approach, emphasizing findings on improving therapeutic strategies and mitigating the burden of fibrotic diseases.

## MATERIALS AND METHODS

### Animal experiments

All experiments were performed with approval by the Canadian Council on Animal Care and the animal care committees of the Department of Comparative Medicine at University of Toronto (protocol no. 20011598) and St. Michael’s Hospital (protocol no. AC143). Wild-type male C57BL/6 J mice at 8 to 12 weeks of age were used according to the current guidelines by the American Thoracic Society (*79*). Experimental pulmonary fibrosis was induced using intratracheal intubation of bleomycin at 0.04 U per mouse in a volume of 50-μl sterile saline (Hospira Healthcare Corporation, NDC 61703-332-18); control animals received only saline (*9*). Animal groups were euthanized after 7 or 21 days. To collect lung tissue, the lungs were cannulated, excised, and washed with phosphate-buffered saline (PBS). For histological analysis, the left lung was removed and inflated to 30 cm of water (cmH_2_O) for 3 to 5 min in 10% formalin solution and fixed for 48 to 72 hours before paraffin embedding.

### Cell culture and drugs

Primary mouse lung fibroblasts and bone marrow–derived monocytes were isolated from 8- to 10-week-old C57BL/6 mice (Charles River Laboratories, Canada), both male and female. Normal mouse lungs were excised, digested in collagenase IA (2 mg/ml; C2674, Sigma-Aldrich, St. Louis, MO, USA), and passed through a 40-μm nylon mesh strainer. The resulting cell suspension was centrifuged for 5 min at 400*g*, and the pellet was resuspended in DMEM (Life Technologies, Burlington, ON, Canada), supplemented with 10% fetal bovine serum (Wisent Bioproducts, St. Jean-Baptiste, QC, Canada), and 1% penicillin/streptomycin (Wisent Bioproducts). Fibroblasts were grown for one to three passages on tissue culture plastic dishes for regular culture maintenance. In selected experiments, fibroblasts were seeded onto silicone elastomer substrates (Excellness Biotech SA, Lausanne, Switzerland), with stiffness matched to normal lung tissue (0.2 to 5 kPa) (*30*) to maintain low mechanical myofibroblast activation levels, or on glass coverslips for live imaging. All experimental substrates were coated with gelatin (2 μg/cm^2^; G9391, Sigma-Aldrich).

Primary Mϕ were obtained by flushing the bone marrow from the femur and tibia of mice and cultured for 5 days in DMEM and F-12 media supplemented with 10% fetal bovine serum, 1% penicillin/streptomycin and M-CSF (20 ng/ml; 315-02, PeproTech, Cranbury, NJ, USA) (fig. S1). Following MSCF treatment, Mϕ were polarized into proinflammatory M_LPS_ using LPS (1 ng/ml; Sigma-Aldrich) and into profibrotic or anti-inflammatory M_IL-4/13_ using combined treatment with IL-4 (10 ng/ml; 214-14, PeproTech) and IL-13 (10 ng/ml; 210-13, PeproTech) for 48 hours, respectively (fig. S1).

To induce fibroblast contractions as a positive control, TRAP6 (10 μM; AS-24190-25, Anaspec Inc., Fremont, CA, USA) was added to monocultures of fibroblasts at the same time Mϕ were added to coculture conditions. As a positive control of Smad signaling, TGF-β1 (2 ng/ml; 299-LT-005, R&D Systems, Minneapolis, MN, USA) was added to monocultures of fibroblasts at the same time Mϕ were added to coculture conditions. To inhibit SACs, GsMTx4 (10 μM; HY-P1410, MedChemExpress Co., Monmouth Junction, NJ, USA) was added to monoculture of fibroblasts for 10 min before lifted and resuspended in fibroblast media without inhibitor present and seeded for coculture experiments. To elicit Ca^2+^ response through Piezo1 in fibroblasts, Yoda1 (30 μM; F14201, Invitrogen, Waltham, MA, USA) was added to fibroblasts stained with Fluo-4 followed by of ionomycin (3 μM; I0634, Sigma-Aldrich). An equal volume to Yoda1 of dimethyl sulfoxide (D8418, Sigma-Aldrich) was used as control. To inactivate αvβ3 integrins, cyclo(-RGDfK) (1 μM; S7834, Selleck Chemicals, Houston, TX, USA, S7834) or Axum4 [1:1000; a gift from D. Sheppard (*45*), University of California, San Francisco, USA] was added to monocultures of M_IL-4/13_ for 2 hours at 37°C. M_IL-4/13_ were lifted and resuspended in fibroblast media without inhibitor present and added to fibroblasts for coculture experiments.

### Contraction assays and analysis

To quantify global fibroblast contraction, we used FLECS assays with cross-shaped patterned substrates (70 μm diagonal; 10-μm bar thickness) in a 96-well plate format, adsorbed with fibronectin (30 μg/ml) and Alexa Fluor 647–conjugated fibrinogen solution (30 μg/ml; a gift of I. Pushkarsky) (*34*). FLECSs were prepared following company procedure (Forcyte Biotechnologies, Los Angeles, CA, USA), and fibroblasts were seeded at a concentration of 4000 cells per well for 30 min before imaging at a frame rate of 3 min using inverted fluorescent microscopy (Cell Discoverer 7, Zeiss) for 2.5 hours in environmentally controlled conditions (37°C and 5% CO_2_). Mϕ, control media, or compounds were added 15 min after starting imaging to establish baseline fibroblast contraction behavior. Mϕ contact–induced fibroblast contractions were quantified by measuring area reduction of the adhesive pattern from binarized image frames using ImageJ (US National Institutes of Health, MD, USA). First, regions of interest (ROIs) of the pattern were analyzed and then overlaid onto a binarized 4,6-diamidino-2-phenylindole (DAPI) frame. Pattern ROIs where no DAPI signal was found were marked in blue (no cell), while ROIs with a DAPI signal were marked in red (cell), to filter out patterns where no cell is present and therefore no contractions occur (Fig. 3A).

To quantify local fibroblast contractions with PIV, collagen gels of 60- to 70-μm thickness were produced on ibidi μ-Dish 35-mm low dishes (80136, ibidi, Gräfelfing, Germany), using pepsin-treated, bovine dermal type I collagen [type I collagen (6.0 mg/ml); 5010 Advanced BioMatrix, San Diego, CA, USA]. Collagen solutions were diluted to a final concentration of 2 mg/ml, neutralized with 0.1 M NaOH to pH 7.4, 125 μl added to an 8.8-cm^2^ surface area, and polymerized at 37°C in 5% CO_2_ for 60 min. To provide markers at the surface of collagens for the measurement of ECM displacement, fluorescent beads (FluoSpheres sulfate, 1.0 μm red, 580/605 nm; F8851, Invitrogen) were diluted to a concentration of 3% in EDAC [1-ethyl-3-(3-dimethylaminopropyl)carbodiimide; 100 μg/ml]. This solution was sonicated for at least 5 min before being added to the surface of collagens and incubated at 37°C for 10 min. The excess bead solution was discarded, and gel surfaces were washed twice with PBS. As published previously, fibroblasts were then seeded sparsely (100 cells/cm^2^) onto the collagen gel surface for 1 hour before starting imaging (*17*). Images were acquired at a frame rate of 3 min using an inverted fluorescence microscope (Zeiss Axiovert 200 M, 40× air, Zeiss, Oberkochen, Germany) for 80 min in environmentally controlled conditions. Mϕ or control medium was added 20 min after starting recording to establish baseline fibroblast contraction behavior. Mϕ contact–induced fibroblast contractions were observed and quantified through changes in fibroblast contraction velocity computed using PIV (*17*). Image stacks were aligned using ImageJ plugin Linear Stack Alignment with scale-invariant feature transform (https://imagej.net/Linear_Stack_Alignment_with_SIFT). All images were then compared one by one to the initial image using the PIV plugin in ImageJ (https://sites.google.com/site/qingzongtseng/piv#tuto) to map bead displacement over time. The bead displacement was then converted to cell traction force using the Fourier Transform Traction Force plugin in ImageJ (http://sites.imagej.net/Template_Matching/) (*80*).

Local cell retractions were quantified for fibroblasts seeded on coverslips for 1 hour, to allow circular spreading, before adding control medium or Mϕ for an additional hour. Following immunofluorescence staining and imaging, binary images of cells were measured for circularity (circle = 1) and solidity (solid object = 1; irregular boundaries or holes <1) using Eq. 1

$$\text{Circularity}=\frac{4\times \text{pi × Area}}{{\left(\text{Perimeter}\right)}^{2}}$$ (1)

The circularity allows us to identify how round the object is, meaning that values closer to 1 are closer to a perfect circle

$$\text{Solidity}=\frac{\text{Area}}{\text{Convex area}}$$ (2)

The solidity (Eq. 2) allows to identify how convex the object is, meaning that the closer the values are to 1, the more the object curves outward; the farther from 1, the greater the concavity. This is achieved by dividing the measured area of the object by its convex area (or convex hull)—accomplished by measuring the convex perimeter through a sequenced measurement around eight Ferets tips (straight lines between two tangents) (*81*). In the areas of retraction of the fibroblast, it creates concavity of the membrane, therefore decreasing the fibroblasts’ solidity.

### *Immunofluorescence microscopy*, *Ca^2+^ imaging*, *and gap junction functional assays*

For immunofluorescence staining of tissue sections, tissue antigens were retrieved by boiling in tris buffer [10 mM tris, 1 mM EDTA (pH 9), and 0.05% Tween 20] at 95° to 100°C for 20 min. After cooling, sections were rinsed in tris-buffered saline (TBS) with 0.025% Triton X-100, blocked with 10% goat serum, and 1% bovine serum albumin (BSA) in TBS for 1 hour, and then stained with primary and secondary antibodies in 1% BSA in TBS. For immunofluorescence microscopy of cultured fibroblasts and Mϕ, cells were fixed with 3% paraformaldehyde, permeabilized with 0.2% Triton X-100, and incubated with primary antibodies directed against F4/80 (GTX26640, GeneTex, Irvine, CA, USA), YAP (sc-271134, Santa Cruz, Dallas, TX, USA), NFAT1 (4389S, Cell Signaling Technology, Beverly, MA, USA), p-MLC (3675S, Cell Signaling Technology), and β3 (CD61) (Axum-4), followed by incubation with secondary antibodies and phalloidin–Alexa Fluor 647 (A30107, Invitrogen) to stain F-actin and DAPI (32670, Fluka, London, UK) to stain nuclei. Images were acquired with Zeiss Axio Observer 7 inverted confocal microscope equipped with LSM 800 scan head and ZEN software (Zeiss, Oberkochem, Germany). Plan-Apochromat (PApo) objectives were as follows: 20×, PApo 20×/0.8; 10×, PApo, 10×/0.45; 40×, numerical aperture (NA) 1.4, oil–differential interference contrast (DIC); and 63×, NA 1.4, oil-DIC (Zeiss).

For live cell video microscopy, fibroblasts were seeded onto glass coverslips and positioned within a Chamlide MB multihole bottom plate “Spaceship” device (CM-B18-1, Live Cell Instruments, Republic of Korea). To reduce fluorescence background and maintain stable pH during recording, we used serum-free, phenol red–free medium with 0.2 M Hepes. For SAC experiments, fibroblasts were stained with 2 μM Fluo-4 AM (F14201, Invitrogen) for 20 min at 37°C. For general Ca^2+^ imaging, fibroblasts were transfected with 1 μg of GCaMP6s plasmid (*82*) (a gift from S. Grinstein, Hospital for Sick Children, Toronto). For live cell imaging of TAZ, a TAZ-citrine plasmid construct (a gift by A. Kapus), prepared as previously described (*83*), was transfected using FuGENE HD transfection reagent (E2311, Promega, Madison, WI, USA). Living cells were imaged using an inverted fluorescence microscope (Axiovert 200 M, Zeiss) equipped with an AxioCam HRm camera and ZEN software (Zeiss). A PApo objective was used (Zeiss, 40× air, NA 0.5) in addition to a Fluar objective (Zeiss, 20×, NA 0.75).

All quantitative image analysis was performed using ImageJ (US National Institutes of Health, Bethesda, MD, USA, http://imagej.nih.gov/ij/, 1997–2013) using customized macros. The mean fluorescence intensity (MFI) levels of nuclear and cytoplasmic YAP (in fixed cells) or only nuclear levels for TAZ-citrine (in living cells) were analyzed from microscopy images using ImageJ. A mask of the nuclei was created by thresholding the DAPI channel, which was used to measure nuclear MFI of YAP and TAZ. The same mask was then used to exclude the nucleus region from measurements of cytoplasmic YAP. For quantitative display, the ratio of nuclear/cytoplasmic MFI was plotted for YAP and nuclear MFI for TAZ. The MFI of GCaMP6s-transfected fibroblasts was measured from microscopy images using ImageJ by outlining the fibroblast from phase images in each frame. Figures were assembled in Adobe Photoshop 2015 (Adobe Systems, San Jose, CA). BioRender was used to prepare schematics.

### Membrane tension and AFM measurements

Membrane tension measurement of fibroblasts in contact with Mϕ was performed using Flipper-TR membrane dye (1 μM; CY-SC020, Cytoskeleton, Denver, CO, USA). Before imaging, Flipper-TR was added to fibroblasts at 37°C for 15 min before washed with medium three times. Images were taken by FLIM (Lambert Instruments BV, Groningen, The Netherlands) to measure the lifetime of the Flipper-TR probe. A higher lifetime represents high tension in the membrane. Lucifer Yellow (25573, Cayman Chemical Company, Ann Arbor, MI, USA) dissolved in distilled H_2_O was used as a reference. LI-FLIM 1.2.26 (Lambert Instruments) software was used for lifetime calculations, through a series of images taken per position to define the excited-state decay rate from the sample. The average time of the excited state of the molecule determines the fluorescent lifetime by the system.

For AFM measurements, fibroblasts and Mϕ were seeded onto gelatin-coated 100-kPa stiff silicone substrates for 48 hours to ensure strong adhesion and flattened cells and assessed in phenol red–free medium supplemented with 20 mM Hepes. To measure the cellular stresses around fibroblast and Mϕ in contact, quadratic pyramidal cantilever probes (MLCT-BIO, Bruker, Billerica, MA, USA) with a spring constant of 0.1 N m^−1^ and a resonating frequency of 38 kHz were used to perform indentation force-distance measurements using the quantitative imaging mode of a NanoWizard 4 system (JPK, Bruker). All tips were calibrated in liquid with contact-based and contact-free methods according to the manufacturer’s instructions. Set point (2.4 nN), *z*-length (1 μm), and extend speed (5 μm s^−1^) were kept constant. Images (70 μm by 70 μm) were generated by the conversion of probe deflection upon indentation into a *z*-length (height). Force curves were generated for every indentation and analyzed using JPK Data Processing Software (Bruker). Height images were processed in micrometers, and force curves were converted into Young’s modulus values (kilopascals) assuming the established Hertz model, describing the deformation of two perfectly homogeneous surfaces. This is estimated by expressing the normal force *F*_n_ of the probe with a radius of *R* as

$$F_{n}=\frac{4}{3}\frac{ER^{1/2}}{\left(1-v^{2}\right)}{\delta_{n}}^{3/2}$$ (3)

where δ_n_ is the indentation depth perpendicular to the sample and v is the Poisson’s ratio of the sample (*84*).

### Scanning electron microscopy and confocal reflection microscopy

For scanning electron microscopy, Mϕ were seeded onto fibroblasts grown on collagen gel–coated 10-mm glass coverslips for 48 hours. Samples were then fixed with 2% glutaraldehyde in 0.1 M sodium cacodylate buffer (pH 7.3) for 2 hours, followed by 0.1 M sodium cacodylate buffer with 0.2 M sucrose (pH 7.3) for 20 min. Samples were dehydrated with ethanol in 10% increments from 50 to 100% for 20 min each and subsequently critical point dried (CPD 030, Bal-Tec, Balzers). Samples were mounted and gold sputtered (Desk II sputter coater, Denton), and images were taken with a scanning electron microscope (FlexSEM1000, Hitachi, Tokyo, Japan) at a tilt angle of 45°.

To visualize collagen fibers in confocal reflectance microscopy, we used a Zeiss confocal microscope (Zeiss). Images were taken with a 40× objective lens in two channels and 57 frames with *z* steps of 1.34 μm, organized as a 3D image through Zen 2.3 lite (Zeiss).

### Western blotting and flow cytometry

For immunoblotting, cells were washed with PBS and lysed with boiling sample buffer [50 mM tris (pH 6.8), 2% SDS, 0.1% bromophenol blue, and 10% glycerol, supplemented with β-mercaptoethanol]. Samples were collected, sonicated, and boiled for 5 min. For coculture experiments, monoculture samples of fibroblasts were mixed with monoculture samples of Mϕ as controls. Protein samples from whole-cell lysis were separated by SDS–polyacrylamide gel electrophoresis, and gels were transferred onto nitrocellulose membrane for immunoblotting. Membranes were blocked for 1 hour at room temperature with blocking solution (1% BSA in TBS) and incubated with primary antibodies overnight at 4°C in blocking solution. Membranes were washed three times for 5 min each with 0.1% Tween 20 in TBS (TBST) and incubated with fluorescent-conjugated secondary antibodies for 1 hour 30 min at room temperature. Secondary antibodies used at 1:5000 dilutions in blocking solutions were anti-rabbit IRDye 680RD and anti-mouse IRDye 800CW (LI-COR Inc.). Membranes were washed three times for 5 min each with TBST and imaged with the Odyssey infrared imaging system (LI-COR Inc.). Integrated fluorescence intensity for each of the Western blot bands was measured from the background-subtracted images. Primary antibodies used were mouse anti-YAP (1:500; H00010413, Abnova, Taiwan, R.O.C) rabbit antiactive YAP (1:500; ab205270, Abcam), rabbit anti–phosphorylated YAP (Ser^127^) (1:1000; 4911, Cell Signaling Technology), and rabbit antivimentin (D21H3) (1:1000; 5741, Cell Signaling Technology).

For flow cytometry, cells were stained using viability dye eFluor780 (1:1500; 65-0865-14, Thermo Fisher Scientific). Cells were live labeled with Brilliant Violet (BV) 421–conjugated F4/80 (1:75; 123132 BioLegend, San Diego, CA, USA), phycoerythrin/cyanine (PE/Cy5)–conjugated F4/80 (1:75; 123110, BioLegend), allophycocyanin (APC)–conjugated CD206 (1:50; 141708 BioLegend), and PE/Cy5-conjugated CD40 (1:75; 124618 BioLegend) for Mϕ polarization validation. Living Mϕ were labeled with APC-conjugated β3 (1:50; CD61, 104315, BioLegend), PE-conjugated αv (1:50; CD51, 104105 BioLegend), and BV421-conjugated CD41 (1:50; 133911 BioLegend) for integrin validation.

To assess direct cell-to-cell communication through gap junctions, calcein-AM (206700 Calbiochem, San Diego, CA, USA) was used as a transfer dye between donor and acceptor cells. Calcein-AM does not emit fluorescence on its own, but once inside live cells, it undergoes hydrolysis by intracellular esterases and transforms into fluorescent calcein. The donor cells were stained with 10 μM calcein-AM for 10 min at 37°C in a 5% CO_2_ environment. A standard curve of calcein-AM in fibroblasts was established to demonstrate the signal of calcein at various concentrations in the fibroblasts. After staining, the donors were lifted using trypsin, centrifuged, and resuspended in culture media. The donor Mϕ were added in a 1:5 (acceptor:donor), and the coculture between donor Mϕ and acceptor fibroblasts was maintained for 1 hour at 37°C in a 5% CO_2_ environment. Cocultured cells were detached using trypsin, centrifuged, and resuspended in a flow buffer (Hanks’ balanced salt solution, 0.1% BSA, and 20 mM EDTA) and stained for F4/80 to distinguish between Mϕ and fibroblast. The staining was performed for 1 hour on ice, washed once with flow buffer, and resuspended in 150 μl of flow buffer. All flow samples were run through a CytoFlex (Beckman Coulter, Mississauga, ON, Canada), gated for live single cells, and analyzed using FlowJo software (Treestar, Ashland, USA).

### Knockdown experiments and quantitative reverse transcription polymerase chain reaction

To knock down Piezo1 (*FAM38a*) and Cx43 (*Gja1*), we used SMARTpool siRNAs (10 nM; Horizon Discovery, Cambridge, UK L-061455 and L-051694, respectively); both targeting siRNAs were compared to a pool of four nontarget siRNAs (10 nM; D-001810 Horizon Discovery). Fibroblasts at 60 to 70% confluency were transfected with Lipofectamine RNAiMAX (13778075 Invitrogen) using a 1:1 ratio of siRNA diluted in Opti-MEM (31-985-070, Thermo Fisher Scientific) and Lipofectamine. Fibroblasts were left at 37°C for 3 to 4 days before use.

For quantitative reverse transcription polymerase chain reaction (PCR), mRNA was extracted by the PureLink Mini RNA Kit (12183018A, Thermo Fisher Scientific) according to the manufacturer’s instructions. RNA (500 ng) was reverse transcribed with the SuperScript VILO cDNA Synthesis Kit (100011932, Invitrogen). PCR amplification was performed in triplicate by RT2 SYBR Green ROX (Qiagen, Redwood City, USA) by using StepOnePlus Real-Time PCR System (Applied Biosystems) at 95°C for 10 min, 40 cycles at 95°C for 15 s, all at 59°C annealing temperature for 60 s followed by the melt curve. Relative gene expressions were calculated by using mouse *Gapdh* as a reference gene and the following mouse-specific primers:

*Ccn1* (AAGAGGCTTCCTGTCTTTGGC; TCGTGTGGAGATGCCAGTTC), *Ccn2* (GAACTGTGTACGGAGCGTGA; CTTCCAGTCGGTAGGCAGC), *Ankrd1* (GTTCCAGGGGTTCATCCACAA; TTTTTGCCTGTTACCAGCTCC), *Acta2* (CATCACCAACTGGGACGACA; GTTCAGTGGTGCCTCTGTCA), *Gapdh* (AGGTCGGTGTGAACGGATTTG; TGTAGACCATGTAGTTGAGGTCA), *Piezo1* (GGCTGTCACTGAGAGGATGTTCA; AGCCACAGCGGATCTGGTA), *Piezo2* (GCCATGCCAAAGTCAATGGTC; TGATTTTCATTGGGCAAGGAGC), and *Gja1* (AGTACCCAACAGCAGCAGAC; TCTGGGCACCTCTCTTTCAC).

### Statistical analysis

GraphPad Prism 10.02 was used for all statistical analyses. All data are shown as individual values with statistics done on means ± SD of at least three independent biological repeats unless otherwise indicated. Statistical significance between conditions was evaluated either by repeated measures one-way analysis of variance (ANOVA) or mixed-effects analysis—with Fisher’s least significant difference, two-way ANOVA, or paired *t* test. Simple linear regressions were applied to measure slopes, with 95% confidence intervals of *X* = 0 and *Y* = 0. Values with *P* < 0.05 were considered statistically significant.

## Supplementary Materials

### The PDF file includes:

Fig. S1 Legends for movies S1 to S3 Image Analysis Code
