# Supplementary material for: Acute contact with profibrotic macrophages mechanically activates fibroblasts via αvβ3 integrin–mediated engagement of Piezo1
Source: Sci Adv. 2024 Oct 23;10(43):eadp4726. doi: 10.1126/sciadv.adp4726 (PMC11498225; doi:10.1126/sciadv.adp4726)
Supplement: Supplementary file 1 — Fig. S1 Legends for movies S1 to S3 Image Analysis Code [file sciadv.adp4726_sm.pdf]

Science Advances
AAAS

Supplementary Materials for

Acute contact with profibrotic macrophages mechanically activates fibroblasts via αvβ3 integrin–mediated engagement of Piezo1

Maya Ezzo *et al.*

Corresponding author: Boris Hinz, boris.hinz@utoronto.ca

Sci. Adv. **10**, eadp4726 (2024)
DOI: 10.1126/sciadv.adp4726

The PDF file includes:

Fig. S1
Legends for movies S1 to S3
Image Analysis Code

Other Supplementary Material for this manuscript includes the following:

Movies S1 to S3

Supplementary Materials

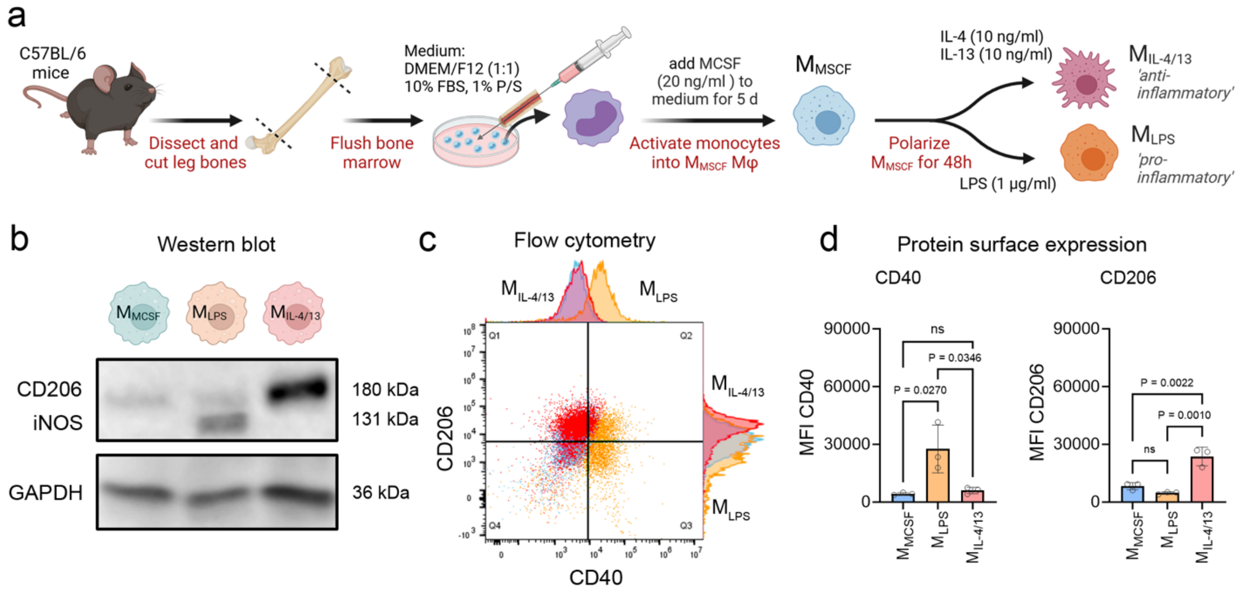

Fig. S1: Murine bone marrow derived Mϕ differentiation validation.

a) Scheme of murine bone marrow derived Mϕ isolation. Bone marrow from C57/BL6 mice flushed from the leg bones of mice. Monocytes treated with MCSF for 5 d to generate Mϕ. Polarization of M_{LPS} with LPS for 48 h and $M_{IL\text{-}4/13}$ with IL-4/13 for 48 h. b) To validate polarization states, key markers for each Mϕ shown through Western blots. Protein level CD206, iNOS, and GAPDH for Mϕ treated with MCSF and LPS or IL-4/13. c) To further validate protein expression, flow cytometry gating of CD206+ versus CD40+ Mϕ is shown. M_{LPS} in orange and $M_{IL\text{-}4/13}$ in red. d) Median fluorescent intensity (MFI) for CD206 and CD40. Statistical significance was calculated using repeated measures ANOVA, and Fisher's LSD post-hoc analysis with significance reached with $P<0.05$.

Movies

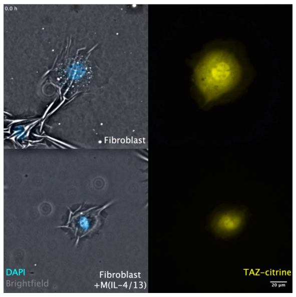

Movie S1: Taz-citrine transfected fibroblasts.

Taz-citrine transfected fibroblasts (yellow) were seeded on 0.2 kPa silicone substrates for 1 h before imaged every 15 min for a total of 3 h; $M_{IL-4/13}$ added after 15 min of imaging. Conditions were fibroblast with control media (+DMEM) or with $M_{IL-4/13}$ contact.

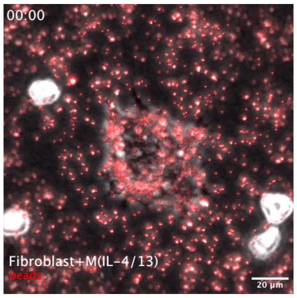

Movie S2: Fibroblast and $M_{IL-4/13}$ on collagen gel laid with red fluorescent beads.

Fibroblasts seeded sparsely (100 cells/cm^2) onto a 2 mg/ml collagen gel with red fluorescent beads on the surface for 1 h before starting imaging at a frame rate of 3 min for a total of 2.5 h.

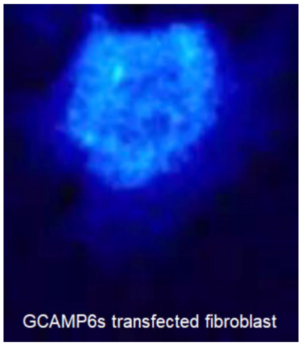

Movie S3: GCaMP6s transfected fibroblast with $M_{IL-4/13}$ contact.

Fibroblasts were seeded on glass coverslips 12 h before being transfected with GCaMP6s plasmid and imaged after another 24 h. Ca^{2+} imaging (1 frame/5 s) of a fibroblast-$M_{IL-4/13}$ contact noted by white arrow.

Image Analysis Code

ROI labeller

```
from fiji.util.gui  import GenericDialogPlus
from ij import IJ, WindowManager, ImageStack, ImagePlus
from ij.measure import  Measurements, ResultsTable
from ij.plugin.frame import RoiManager

RM = RoiManager()
RT = ResultsTable()
group1 = []

#Create GUI for displaying errors
error_gui = GenericDialogPlus("Error!")
error_gui.hideCancelButton()

class Mask():
    '''
    Mask class defines a binary image that can be selected from open images by the user using the get_user_input method
    '''
    def __init__(self):
        self.bin = ImagePlus()

    def get_user_input(self):
        # Create a GUI for selecting the DAPI mask from open files
        gui = GenericDialogPlus("Mask Selection")
        gui.addMessage("Input binary mask for DAPI channels")
        gui.addImageChoice("DAPI Mask","DAPI Mask from thresholded image")
        gui.showDialog()

        if gui.wasCanceled():
            return False
        if gui.wasOKed():
            try: #Checks if image was actually selected
                m = gui.getNextImage()
                if not m.getProcessor().isBinary():
                    raise ValueError #Additional check for if image is a binary image
                self.bin = m
                return True

            except:
                error_gui.addMessage("Error: Invalid mask input")
                error_gui.showDialog()
                return False

def group_ROIs(bin):

    '''
    Function that groups ROI in the ROI manager into two groups based on an input binary mask;
    Group 0 = EMPTY and 1 = Contains signal

    This function uses and clears the default Results Table
    '''
```

```
    for roi in RoiManager.getInstance().getRoisAsArray(): #Cycles through each ROI in the ROI Manager
        bin.setRoi(roi)
        IJ.run(bin, "Measure", "") #Measure the ROI on dapi to see if there are any cells in that ROI

        if RT.getResultsTable().getValue("Max",RT.getResultsTable().size()-1) == 0: #ROIs without cells
are in group 0
            roi.setGroup(1)

        else: #ROIs containing cells are in group 1
            roi.setGroup(2)


    #Clear Results Table

    IJ.selectWindow("Results")
    IJ.run("Close")

if __name__ == "__main__":

    dapi = Mask() #Creates a Mask object for the DAPI channel

    if len((RoiManager.getInstance().getRoisAsArray())) == 0: #Check if the ROI manager is empty
        error_gui.addMessage("Error: No ROIs in ROI manager")
        error_gui.showDialog()
    elif dapi.get_user_input():  #Get DAPI channel mask from user
        group_ROIs(dapi.bin) #Group ROIs using the DAPI channel mask
```
